# Ultra-Wideband Antennas for Biomedical Imaging Applications: A Survey

**DOI:** 10.3390/s22093230

**Published:** 2022-04-22

**Authors:** Umair Rafique, Stefano Pisa, Renato Cicchetti, Orlandino Testa, Marta Cavagnaro

**Affiliations:** Department of Information Engineering, Electronics and Telecommunications, Sapienza University of Rome, 00184 Rome, Italy; stefano.pisa@uniroma1.it (S.P.); renato.cicchetti@uniroma1.it (R.C.); orlandino.testa@uniroma1.it (O.T.)

**Keywords:** microwave imaging, near-field, UWB antennas

## Abstract

Microwave imaging is an active area of research that has garnered interest over the past few years. The main desired improvements to microwave imaging are related to the performances of radiating systems and identification algorithms. To achieve these improvements, antennas suitable to guarantee demanding requirements are needed. In particular, they must operate in close proximity to the objects under examination, ensure an adequate bandwidth, as well as reduced dimensions and low production costs. In addition, in near-field microwave imaging systems, the antenna should provide an ultra-wideband (UWB) response. Given the relevance of the foreseen applications, many UWB antenna designs for microwave imaging applications have been proposed in the literature. In this paper, a comprehensive review of different UWB antenna designs for near-field microwave imaging is presented. The antennas are classified according to the manufacturing technology and radiative performances. Particular attention is also paid to the radiation mechanisms as well as the techniques used to reduce the size and improve the bandwidth.

## 1. Introduction

In recent years, near-field microwave imaging has received a considerable amount of interest, especially for the detection and localization of malignant tissues in the human body [1]. It has been proposed to monitor thermal ablation of liver tumors and cardiac activities, and to detect breast and brain tumors. Microwave imaging, when compared to traditional methods such as mammography [2] and ultrasound [3], offers promising solutions for a variety of biomedical applications [4]. Generally, X-ray mammograms do not provide exact results since the rate of false and negative results has been accounted in the range of 4% to 34% [5]. Such outcomes, which are due to the inherent limitations of X-ray mammography, lead to increased disease-specific mortality and morbidity or unnecessary interventions. Ultrasound imaging is considered an alternative to X-ray mammograms. Like mammograms, ultrasound imaging is a sensing/detection strategy that has some limitations. Although it is a simpler method than mammography, it somehow fails to recognize whether the lesion is benign or malignant.

**Figure 1:**
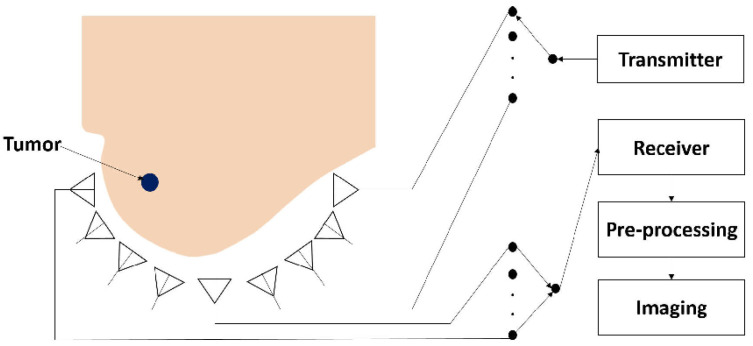
A typical near-field microwave imaging system for breast cancer detection.

Microwave imaging, on the other hand, employs short-pulses of low-power microwaves emitted by suitable antennas towards the human body. Then the antennas collect the backscattered energy, which is processed to create an image. As an example, let us consider a typical breast cancer detection system, such as that shown in Figure 1. Several antennas meant to operate in the near-field region are placed in an array configuration around the breast. The antennas forming the array are sequentially selected to transmit pulses into the breast. Then, the backscattered signals are collected by the receiving antennas. Finally, a suitable signal processing technique is applied to these signals for image reconstruction.

Several reasons suggest the use of microwave imaging for biomedical applications. In particular, the different spectral behaviors of the permittivity and conductivity of healthy and carcinogenic tissue in the microwave range can be exploited to produce a high-quality three-dimensional (3D) image [2]. Furthermore, the non-ionizing transmitted pulses are of low power [1], and therefore, the energy absorbed by the human tissues is low, which does not create potential health risks [6].

This paper presents a comprehensive review of several ultra-wideband (UWB) antenna designs for near-field microwave imaging. The paper is organized into six sections. In Section 2, a brief overview of microwave imaging along with the different approaches used to reconstruct the image is presented. In Section 3, the antenna design challenges for microwave imaging systems are discussed. In Section 4, a detailed analysis of different antenna designs available in the literature, with particular emphasis on those conceived for near-field microwave imaging systems, is presented. In Section 5, an analysis that highlights the advantages and shortcomings of the antennas is provided. Finally, concluding remarks are given in Section 6.

## 2. An Overview on Near-Field Microwave Imaging

Reconstruction methods for microwave imaging have received a lot of interest in recent years. At first, imaging took place in the far-field, necessitating the employment of antennas (waveguide and horn antennas) with high directivity and gain, while operating in the low gigahertz band (1–3 GHz) [7]. The complexity and limitations of diffraction-based methods [8] sparked interest in developing near-field algorithms. Correspondingly, the experimental set-up moved to array of flexible, low cost antennas as those presented in Section 4 of this paper.

**Figure 2:**
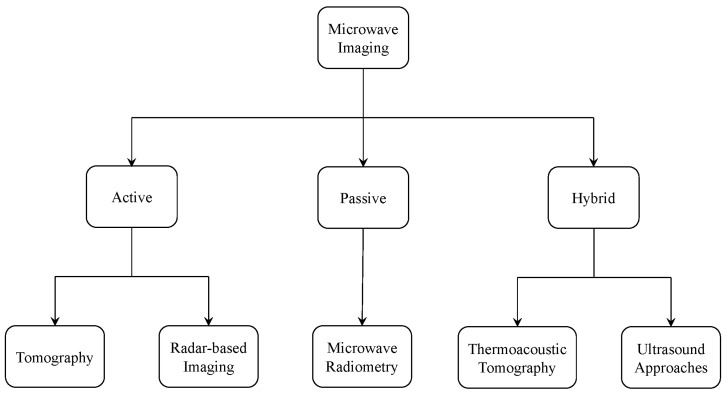
Different approaches used for near-field microwave imaging.

As shown in Figure 2, several approaches have been introduced in literature to reconstruct images of human tissues by way of near-field microwave imaging.

In the active approach, an array of antennas is placed around the human body; the transmitted and reflected electromagnetic signals are collected and used to create images. If a tumor is present, the incident electromagnetic waves will be scattered due to the change in dielectric properties exhibited by the tumor with respect to the surrounding tissues. So, by extracting the information contained in the scattered signals, 3D microwave images can be generated [9]. The active approach can be implemented using two main techniques.

The first technique is transmission-reflection imaging, also known as microwave tomography [10,11,12]. This technique employs a single antenna element to transmit microwave signals into the human body. Then, the transmitted signal is received by several antennas placed on the other side of the human body. This procedure is repeated many times by changing the position of the transmitting antenna. Finally, the received signals are employed to derive the spatial distribution of the human body permittivity by comparing the measured data with simulated ones. These latter are calculated by solving the direct electromagnetic problem with iteratively updated property estimates [13]. This approach requires antennas suitable to operate in the frequency range between 500 MHz and 6 GHz, because higher frequency signals are strongly attenuated in the human tissues, while lower frequencies show low resolution.

The second active approach is reflection imaging, also known as radar-based imaging [14,15,16]. This approach is mainly inspired by radar systems for radio detection. In this technique, antennas placed at different locations are used to transmit short microwave pulses, which are then received by antennas placed at different locations around the human body. From the reflected signals, a complete profile of the dielectric properties of the human tissues can be obtained by synthetically focusing the received signals in different locations of the body. To this end, several algorithms, such as Delay-And-Sum (DAS) [17], Improved Delay-And-Sum (IDAS) [18], Delay-Multiply-And-Sum (DMAS) [19], Channel Ranked DAS (CR-DAS) [20], and Coherence Factor-Based DAS (CF-DAS) [21], etc., can be used. These methods used coherent backscattered radar signals acquired after the human body is illuminated with UWB pulses. In this case, to obtain high-resolution imaging, high-gain wideband antennas are required.

The passive approach is commonly known as microwave radiometry [22,23]. In this approach, it is assumed that the malignant tissue possesses a higher temperature compared to healthy tissues due to the change in dielectric properties. Based on this assumption, the difference in temperature between the normal and malignant tissues is retrieved.

In the hybrid approach, which is based on microwave-acoustic imaging, microwave fields are used to heat the tissues [24]. As a result, pressure-wave signals are generated, which can be measured with ultrasound transducers, and the acquired data are used in image reconstruction.

Besides imaging applications, multi-antenna systems are used in applications where beamforming as well as focusing issues are involved. Examples are hyperthermia applications as well as implantable medical devices [25,26]. In these applications, antenna feeding in terms of amplitude and phase aimed at maximizing the deposition of energy in the region of interest (tumor, implantable devices, etc.) come into play. Since the present paper is focused on near-field microwave imaging, it is suggested to the readers to consult the papers [25,27,28] where suitable algorithms to identify the optimal excitation profiles of multiport networks are reported.

## 3. Antenna Design Considerations

One of the most important components in near-field microwave imaging systems is the antenna, whose design is critical for overall system performance. For near-field microwave imaging systems, the antenna element should provide wide bandwidth and should be able to transmit pulses with little signal distortion. Furthermore, the time-domain antenna performances should be good enough so that the transmitted pulses can penetrate through the human tissues with limited distortions of the signal waveform. To meet these requirements, UWB technology may prove beneficial, since the pulse duration in UWB systems is generally of the order of a few nanoseconds, which results in a bandwidth of some gigahertz.

Some of the performance parameters for the design of an UWB antenna meant for microwave imaging applications are discussed below.

- **VSWR and Reflection Coefficient:** When electromagnetic waves travel from the source to the antenna through the feed line, they may face an impedance mismatch. Due to this, some of the energy is reflected back to the source, producing a standing wave whose level is measured by the voltage standing wave ratio (VSWR). VSWR is defined as *the ratio of maximum to minimum voltage amplitude along the feeding line*. Ideally, it should be equal to one, and it can be calculated as [29,30]:
  (1) $$\mathrm{VSWR}=\frac{1+|S_{11}|}{1-|S_{11}|}$$
  where $|S_{11}|$ corresponds to the amplitude of the antenna reflection coefficient and it is defined as *the ratio of the reflected wave to the incident wave*. Mathematically, it can be calculated as [29,30]:
  (2) $$S_{11}=\frac{Z_{A}-Z_{0}}{Z_{A}+Z_{0}}$$
  where $Z_{A}$ is the antenna impedance and $Z_{0}$ is the characteristic impedance of the feeding line.
  In addition, the antenna band (*B*) is defined as *the frequency range where the magnitude of the reflection coefficient is less than −10 dB* (VSWR < 1.9). Furthermore, the fractional bandwidth (FBW) is defined as *the ratio between B and the central frequency ($f_{c}$) of the antenna band*.
- **Time Domain Performance:** As previously discussed, UWB antennas transmit pulses with a few nanoseconds duration [31,32]. To characterize the time domain performance of UWB antennas, various parameters such as fidelity factor, group delay, and phase response can be employed [33]. To evaluate these parameters, one transmitting and one receiving antenna are placed at a given distance and the transmitting antenna is excited with a Gaussian pulse or by means of derivatives of the same signal [34].
  ○ *Fidelity Factor:* To analyze the time domain pulse distortion, a parameter called fidelity factor may be employed. It is defined as *the maximum amplitude of the cross-correlation between the radiated E-field and the input signal*. Generally, two distinct orientations (face-to-face and side-by-side) are taken into account to calculate fidelity factor. It can be calculated using the following expression [35]:
    (3) $$FF=\max_{\tau }\int_{-\infty }^{\infty }{\hat{T}}_{s}\left(t\right){\hat{R}}_{s}\left(t+\tau \right)d\tau$$
    where
    (4) $${\hat{T}}_{s}\left(t\right)=\frac{T_{s}\left(t\right)}{{\left[\int_{-\infty }^{\infty }{\left|T_{s}\left(t\right)\right|}^{2}dt\right]}^{\frac{1}{2}}}$$
    (5) $${\hat{R}}_{s}\left(t\right)=\frac{R_{s}\left(t\right)}{{\left[\int_{-\infty }^{\infty }{\left|R_{s}\left(t\right)\right|}^{2}dt\right]}^{\frac{1}{2}}}$$
    where $T_{s}\left(t\right)$ and $R_{s}\left(t\right)$ correspond to the transmitted and received pulse, respectively. The value of the fidelity factor is in the range between 0 and 1. When it is equal to 1, the received signal pulse has the same shape as the input signal pulse. If it is equal to 0, then the received signal pulse is completely different from the transmitted one. It has been found that when the fidelity factor is equal to or below 0.5, it is difficult to recognize the received pulse.
  ○ *Group Delay and Phase Response:* Another important parameter used to characterize the time domain antenna response is the group delay, which is useful for estimating the radiated field signal distortion. In fact, group delay peaks highlight the presence of resonant processes that take place in the antenna structure and which are responsible for the ringing in the time domain antenna response. The group delay is defined as *the negative derivative of the signal phase response with respect to frequency* and it can be evaluated as [35]:
    (6) $$\tau_{g}\left(\omega \right)=-\frac{d\phi (\omega )}{d\omega }$$
    where ϕ denotes the phase response of the antenna and ω represents the angular frequency.
    Group delay should be constant, or its deviation should be less than 1 ns within the antenna operating frequency band, to ensure pulse transmission with limited signal distortion [35].
- **Radiated Electromagnetic Field Distribution:** The antenna far-field parameters, such as radiation pattern, directivity, −3 dB beam aperture, etc., are not valid in the near-field and, as such, they should not be used to evaluate antennas’ performances when near-field applications are considered. In this regard, other parameters were proposed in the literature, such as the half energy beamwidth (HEBW), defined as *the width of the area in which the energy is greater than or equal to half the maximum value on a plane orthogonal to the antenna aperture* [36]. However, several papers are available in the literature in which the authors present antenna designs for near-field imaging applications even though they show the far-field region antenna parameters.
- **Feeding of the Antenna:** Most of the antennas discussed in this paper present a planar design, and as such are fed through a microstrip or a coplanar line. However, typically, signals are generated or received in coaxial technology, so that a coaxial to microstrip connector is to be used. In such a case, the presence of the connector should be taken into account in the design process, since it could introduce mismatches in the feeding line [37].

In addition to the above-mentioned requirements, in medical applications, the antenna size should be small enough so that it would occupy a small area with respect to the body region being investigated. On the other hand, as already mentioned, the antenna is used in the near-field and it should radiate a field which is able to penetrate the human body, so this imposes several challenges. Both aspects will be discussed in the following sub-sections.

### 3.1. Coupling Medium

To maximize the field excited within the human body it is necessary to place the antennas very close or in direct contact with the body, which results in reflections occurring at the antenna-air-skin interfaces. This issue can be limited by putting the antenna inside a coupling medium whose dielectric properties are close to those of human tissues. However, the coupling medium has its effects on antenna performance. In fact, its presence causes an alteration of the antenna current distribution, and consequently, the input impedance and the radiation properties of the antenna will be affected. In addition, a coupling medium is responsible for an additional field attenuation between the source and the observation point. Therefore, when designing an antenna for microwave imaging systems, the presence of the coupling medium must be taken into account directly at the design stage so that the performance of the antenna and the imaging system does not deteriorate too much [37].

### 3.2. Electromagnetic Field Penetration in Biological Tissues

To detect a tumor, or in general, to perform an image of the human body, the electromagnetic field radiated by the antenna should be able to reach the target (tumor) and come back to the receiving antenna. As a first approximation, the power attenuation for this round trip is: A(dB)=2×8.681αL, where *L* is the depth of the target within the tissue and the attenuation constant (α) is given by

(7) $$\alpha =\omega \sqrt{\mu_{0}\epsilon_{0}}\sqrt{\frac{\epsilon_{r}^{\prime }}{2}\left[-1+\sqrt{1+{\left(\frac{\sigma }{\omega \epsilon_{0}\epsilon_{r}^{\prime }}\right)}^{2}}\right]}$$

where $\mu_{0}$ and $\epsilon_{0}$ represent permeability and permittivity of free space, respectively, and $\epsilon_{r}^{\prime }$ denotes the real part of the complex relative permittivity ($\epsilon_{r}=\epsilon_{r}^{\prime }-j\epsilon_{r}^{\prime \prime }$) of biological tissues and $\epsilon_{r}^{\prime \prime }$ represents the imaginary part of the complex relative permittivity. In Equation (7), σ (S/m) is the equivalent conductivity, including both static conductivity and dielectric losses ($\epsilon_{r}^{\prime \prime }$). In the following pages, when reporting the dielectric properties of substrate materials, which are non-dispersive in nature, the used symbol will be $\epsilon_{r}$, being evident that in this case the relative permittivity reduces to a real number. Table 1 lists $\epsilon_{r}^{\prime }$ and σ of different biological tissues at different frequencies.

Since, the received power in dB is equal to the transmitted power in dB minus the attenuation in dB, for an imaging system to operate correctly, the received power must be greater than the sensitivity of the receiver.

## 4. UWB Antennas for Near-Field Microwave Imaging

This section reviews several UWB antenna designs intended for microwave imaging applications. These antennas are divided into two main categories: planar and non-planar antennas. Planar antennas are further classified according to their electromagnetic behavior, such as traveling wave antennas (Vivaldi antennas) (Section 4.1), planar monopole antennas (Section 4.2), slot antennas (Section 4.3), and bowtie antennas (Section 4.4). Non-planar structures are classified into two types: horn antennas (Section 4.5) and dielectric resonator antennas (DRAs) (Section 4.6). The investigated antenna’s properties are described in terms of enhanced bandwidth, radiation characteristics, and miniaturized design.

### 4.1. Vivaldi Antennas

**Figure 3:**
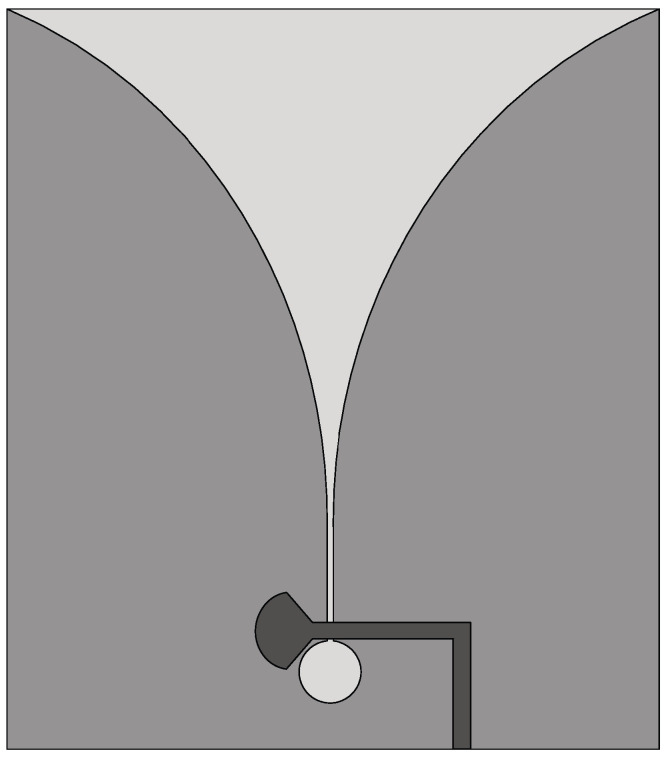
Basic design structure of Vivaldi antenna (redrawn from [40]). Dark gray top metal face, medium gray bottom metal face, and light gray dielectric substrate.

The Vivaldi antenna is one of the most suitable candidates for applications where broadband behavior and directional characteristics are required. It was first introduced in 1979 by Gibson [39] and belongs to the class of slow end-fire traveling-wave antennas. The basic structure of a Vivaldi antenna consists of an exponentially tapered planar slot, usually fed through electromagnetic coupling from a microstrip feeding line, as shown in Figure 3.

In the Vivaldi antenna, the non-resonant radiation mechanism is produced by waves traveling along the tapered radiating slot of the antenna [39]. The traveling wave energy is tightly confined between the slot conductors when the distance between them is very small, with respect to the wavelength, while it is scattered in space by the radiating slot when the distance between the two conductor arms increases [39]. The main requirement for gain is that the phase velocity of the bounded wave is equal to or greater than that of the surrounding medium, which requires continuous compensation of the initial phase of the traveling wave field.

With these antennas, FBW of 100% can easily be achieved together with a maximum gain in the range of 5–8 dBi [41,42]. Finally, the typical fidelity factor is about 90% [40]. Due to their broadband behavior, high gain, and directional radiation characteristics [43], Vivaldi antennas have been employed in many applications, including microwave imaging systems for biomedical [36] and through-the-wall applications [42].

#### 4.1.1. Techniques Employed to Enhance the Bandwidth of Vivaldi Antennas

**Figure 4:**
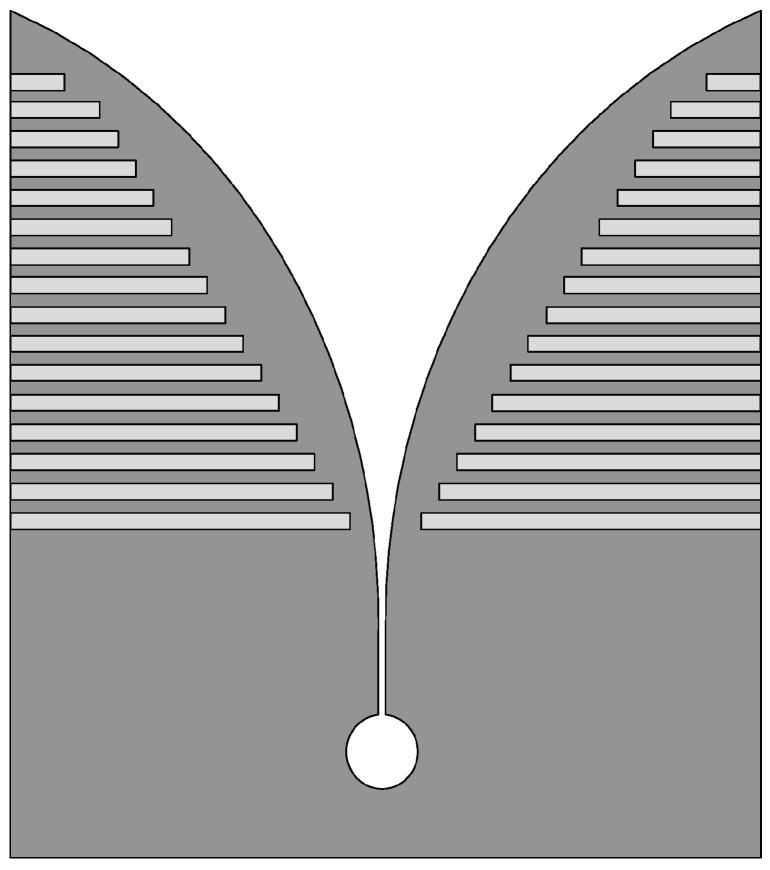
Schematic of the corrugated Vivaldi antenna (redrawn from [45]). Medium gray metal face and light gray slots.

Vivaldi antennas with suitable corrugations on the radiating arms exhibit a wider impedance bandwidth and a higher gain compared to the basic Vivaldi antenna. The corrugated structure modifies the configuration of the currents along the outer edges of the metal surfaces forming the antenna, thus better confining the field in the region of the radiating slot [44]. In [45], a corrugated Vivaldi antenna design was presented for wideband microwave imaging applications. By introducing suitable corrugations on the external edges of a conventional Vivaldi antenna arms, as shown in Figure 4, a wideband response from 2 GHz to 8 GHz (FBW = 120%) was observed with improved radiation characteristics.

**Figure 5:**
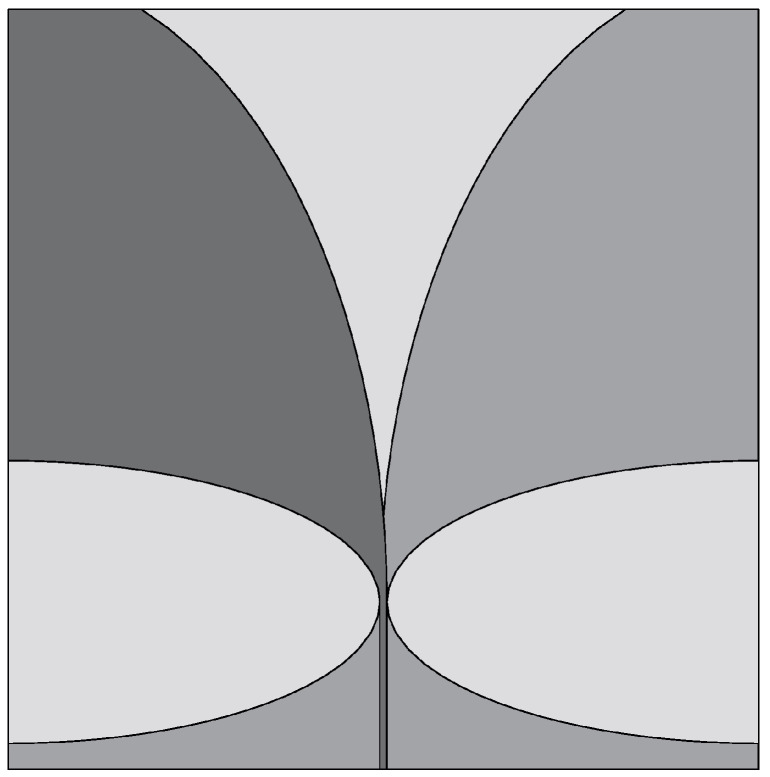
Design of exponentially TS-AVA (redrawn from [47]). Dark gray top metal face, medium gray bottom metal face, and light gray dielectric substrate.

Gazit [46] presented a new design of the tapered slot antenna (TSA), named the antipodal Vivaldi antenna (AVA). In this design the two arms of the radiator are etched on the two opposite sides of the substrate. The bandwidth of TSA can be enhanced by using the AVA because of its feeding technique, which is based on a transition between a microstrip line to double-sided parallel stripline. This ensures the in-band antenna entry at a lower frequency. In AVA, both the inner and outer edges of the metallic arms fit exponential curves. Kanjaa et al. [47] designed the exponentially tapered slot antipodal Vivaldi antenna (TS-AVA), shown in Figure 5, for breast cancer detection. Asymmetric exponential tapered slots were etched from the conventional AVA to achieve impedance bandwidth in the frequency range of 4–12 GHz (FBW = 100%).

**Figure 6:**
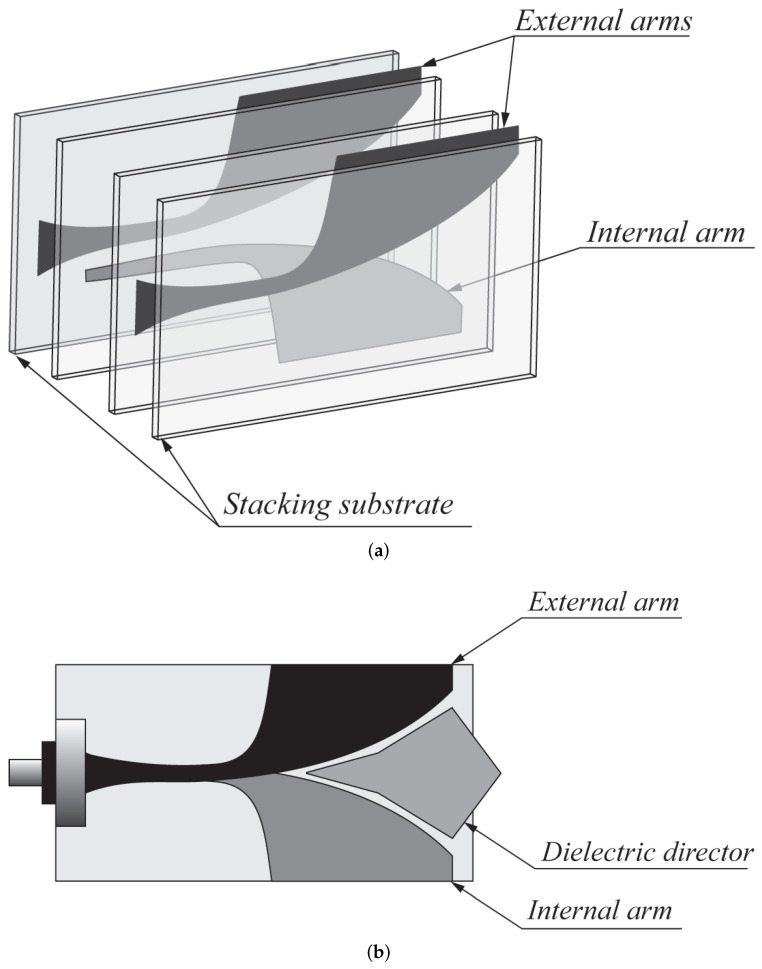
(**a**) Multi-layer BAVA design (redrawn from [36]) (**b**) BAVA with dielectric reflector (redrawn from [49,50]). Black external arm, dark gray internal arm, medium gray dielectric director, and light gray dielectric substrate.

Langley et al. [48] introduced a different Vivaldi antenna, which is known as balanced AVA (BAVA). This antenna has a wide bandwidth and it also provides a reduced level of cross-polarization, thanks to a higher geometric symmetry, compared to AVA. The main disadvantages of BAVA are its high cost and complex design. In [36], a multi-layer BAVA design was presented for a tissue sensing adaptive radar system. Three copper layers were used for the antenna design, as shown in Figure 6a. The top and bottom layers were connected to the feed line ground planes, and the middle layer was connected to the signal layer. The copper layers were supported by two dielectric substrates and two additional dielectric substrates were utilized on each side of the antenna to equalize the frequency behavior of the gain and antenna main beam. The measured results showed that the antenna operates from 2.4 GHz to 12 GHz (FBW = 133.33%).

In [49,50], the authors employed a dielectric director (acting as a dielectric lens) in a BAVA (see Figure 6b) to enhance the directivity of the antenna. They demonstrated that the dielectric-based BAVA provides narrower beamwidth and a wide impedance bandwidth ranging from 2.4 GHz to over 18 GHz (FBW = 153%). The antenna presented in [50] was designed to operate in a coupling medium (canola oil) with the aim of detecting tumors more efficiently.

**Figure 7:**
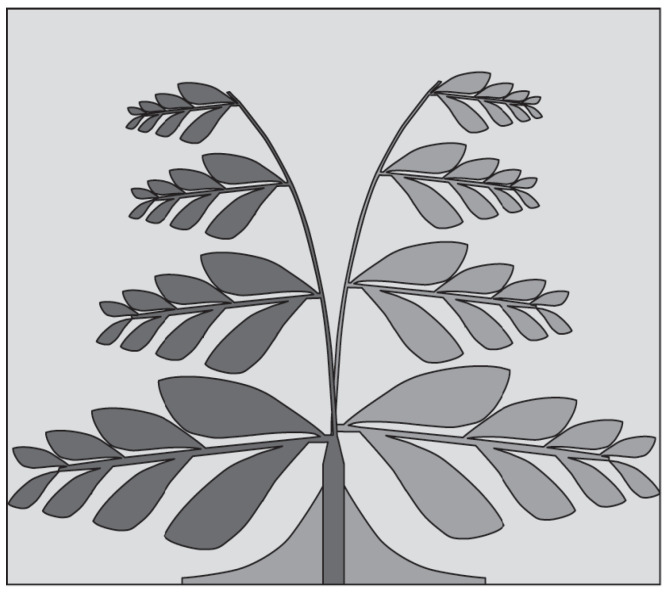
Fen leaf inspired fractal AVA for microwave imaging applications (redrawn from [56]). Dark gray top metal face, medium gray bottom metal face, and light gray dielectric substrate.

A wide impedance bandwidth can also be achieved by utilizing self-likeness characteristics of fractal geometry [51]. Fractals are generally unique structures that can be realized using iterative recursive procedure. The most commonly used and well-known fractal geometries are Minkowski [52], Koch [53], Hilbert curve [54], and Sierpinski Gasket/Carpet [55]. Researchers have utilized these fractal geometries to realize antennas for wireless and biomedical applications. In [56], a fern leaf-shaped fractal AVA design was presented for wideband microwave imaging systems (see Figure 7). With the use of fractal geometry, about 60% size reduction was achieved in Vivaldi antenna dimensions compared to conventional AVA. Although the design was complex, it demonstrated an impedance bandwidth of about 18.7 GHz from 1.3 GHz to 20 GHz (FBW = 175.58%).

**Figure 8:**
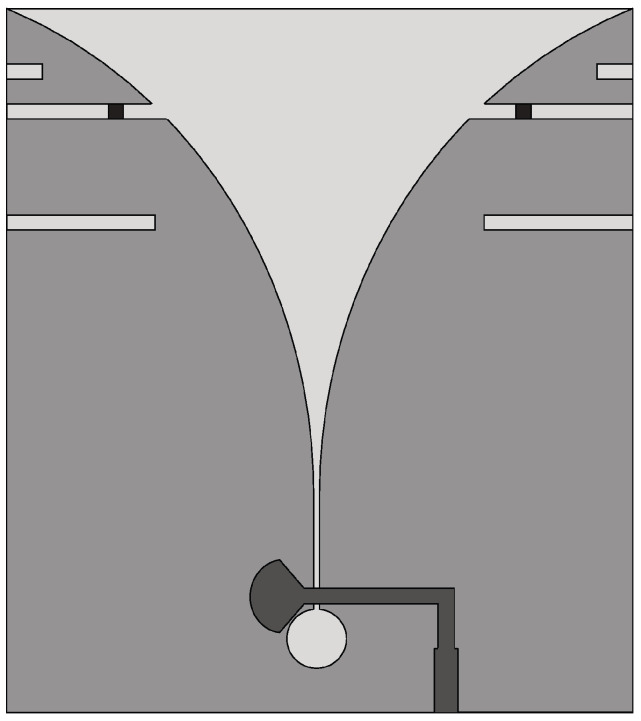
Front view of the resistance-loaded dual-layer Vivaldi antenna (redrawn from [57]). Dark gray top metal face, medium gray bottom metal face, black resistance, and light gray dielectric substrate.

Resistive loading is another technique that has shown its ability in enhancing the impedance bandwidth of Vivaldi antennas even if it reduces their energy efficiency. Zhao et al. [57] designed a resistance-loaded dual-layer Vivaldi antenna for plasma reflection diagnosis applications. The designed antenna consisted of five parts: a dual-layer Vivaldi, a feeding strip, an SMA-K connector, and a double-layer substrate (see Figure 8). The feeding strip was sandwiched between two layers of Vivaldi to form a stripline structure. Four 100 Ω chip resistors were added to the Vivaldi antenna arms to reduce its size and improve the reflection coefficient at low frequencies. Simulation and measurement results showed that the resistive Vivaldi antenna operates well in the frequency range 2–16.3 GHz (FBW = 156.28%).

#### 4.1.2. Techniques Useful to Enhance Vivaldi Antenna Radiation Characteristics

Fractal geometries have also been utilized to improve the radiation characteristics of the Vivaldi antenna. In [58], a palm tree-like coplanar Vivaldi antenna was designed for near-field radar imaging applications, to operate in L- and S-band (1–4 GHz). The performance of the proposed antennas was compared with that of conventional Vivaldi and AVA, and it was demonstrated that the presented antenna designs offered a maximum gain of 10.74 dBi in the operating range with respect to 7.0 dBi for the conventional structure.

One of the techniques to improve the radiation properties of the conventional Vivaldi and AVA is to employ different slot shapes on the metallic arms of the radiators. For example, in [59], a Koch fractal slot-loaded AVA was designed for microwave imaging applications. It was demonstrated that the adoption of the Koch fractal slot extends the current path, improving the lower band frequency entry, while at the same time reducing sidelobe levels (SLLs), and increasing the gain. The operating frequency range of the designed AVA is from 4.87 GHz to 11 GHz where the antenna exhibits a constant gain (>8 dB). Furthermore, the designed antenna can detect brain tumor with high accuracy and provides high contrast imaging compared to conventional AVA.

**Figure 9:**
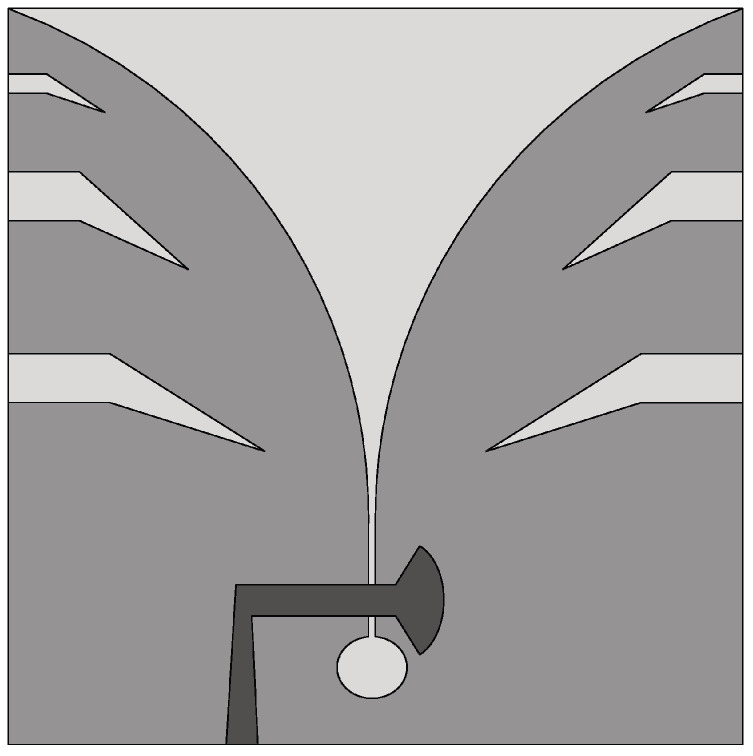
Geometrical layout of side slot-loaded Vivaldi antenna (redrawn from [60]). Dark gray top metal face, medium gray bottom metal face, and light gray dielectric substrate.

A design of a side slotted Vivaldi antenna (SSVA) was presented in [60] for cancer detection. To increase the electrical length of the antenna, irregular slots were etched from the edges of the radiating arms, as shown in Figure 9, which ultimately reduced the lower operating frequency of the antenna. A bandwidth of about 5.46 GHz was achieved from 1.54 GHz to 7 GHz with a peak gain of 9.8 dBi. The fidelity factors for face-to-face and side-by-side configurations were 98% and 44.79%, respectively.

**Figure 10:**
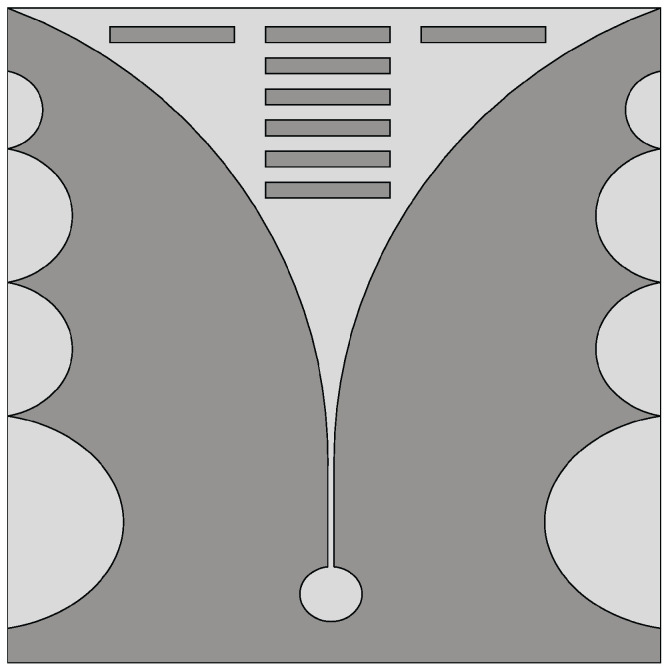
Schematic of the hemi cyliderical slots-based Vivaldi antenna with planar directors (redrawn from [61]). Medium gray bottom metal face and light gray dielectric substrate.

In [61], a semicircular slots-loaded UWB Vivaldi antenna was designed and presented. Four semicircular slots of decreasing radius were etched from the edges of the radiating arms of the Vivaldi antenna, shown in Figure 10, to increase the antenna’s electrical length and transfer maximum energy towards the slot center. The antenna was designed on a 0.8 mm thick FR-4 substrate with an overall size of 49 mm × 48.4 mm. An operational bandwidth from 2.9 GHz to 10.4 GHz was observed. Moreover, planar directors were added in front of the Vivaldi antenna aperture to enhance its radiation characteristics (see Figure 10). Using these directors, a peak gain of about 9.58 dBi was achieved in the operating frequency range. Finally, a fidelity factor of 95.57% was observed, which proves the low distortion of the transmitted pulses.

**Figure 11:**
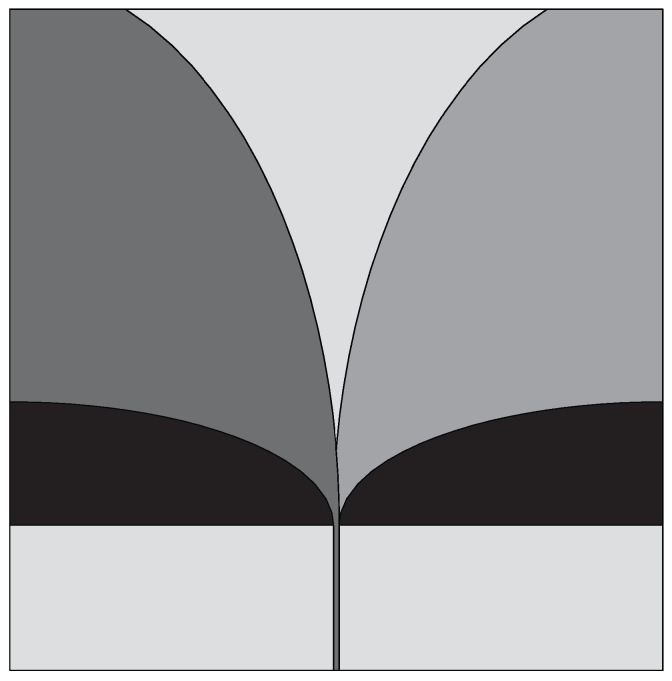
Resistive layer-based AVA for UWB microwave imaging systems (redrawn from [62]). Dark gray top metal face, medium gray bottom metal face, black resistive metal face, and light gray dielectric substrate.

In [62], a design of conventional AVA was presented for UWB medical imaging systems. The authors utilized resistive layers on the front and back sides of the antenna, as shown in Figure 11, to improve directivity and minimize back radiations. The designed antenna operates in the UWB frequency range (3.1 to more than 10.6 GHz) and offers directional radiation characteristics with a peak gain between 4 dBi and 11 dBi. Moreover, the designed antenna offers a fidelity factor of more than 90% when operating in free space, which decreases up to 80% when the signal propagates inside the human body.

An antenna structure producing a circular polarization can be obtained by using the cross configuration of Vivaldi antennas. In [63], a cross-Vivaldi antenna design was presented for tumor detection using cross-polarization reflections. The designed antenna consisted of two identical Vivaldi elements placed in a cross position through suitable cuts realized into each antenna element. A metallic plate was placed behind the antenna elements to block the backward radiation. To optimize tumor detection, the antenna was placed in a coupling medium and one Vivaldi element was used to illuminate the object, while the other one received the reflected signals. The designed antenna operates in the frequency range of 2.2–5.4 GHz.

#### 4.1.3. Techniques Employed to Reduce Vivaldi Antenna Dimensions

**Figure 12:**
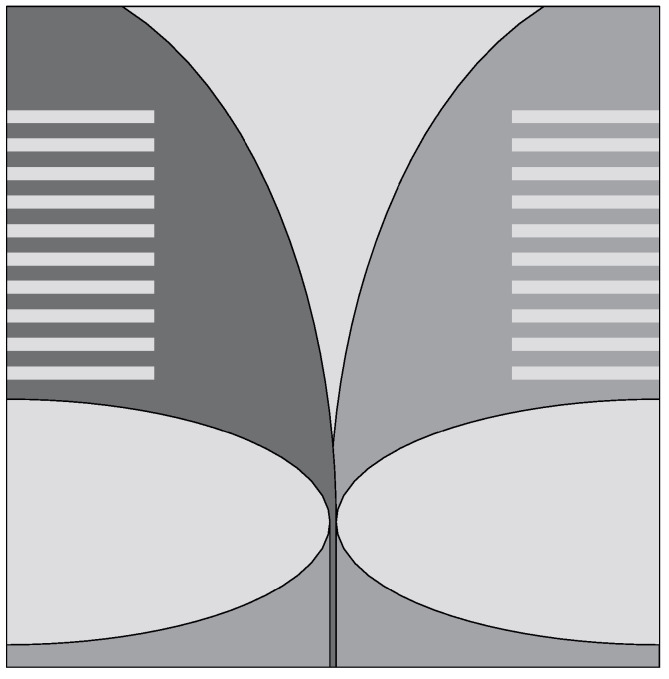
Schematic of the corrugated AVA design (redrawn from [64]). Dark gray top metal face, medium gray bottom metal face, and light gray dielectric substrate.

The use of corrugations may also help to reduce the size of an antenna. An enhanced bandwidth corrugated AVA design was presented in [64] (see Figure 12). The corrugations were introduced in the top radiator and the bottom ground, and their lengths and widths were optimized to reduce the antenna’s size and improve the antenna’s matching. By using this technique, an overall size of 22 mm × 40 mm was realized. The introduction of corrugations suppresses standing waves arising in the antenna, which leads to improved directivity for lower frequency bands as well as enhanced transmission of UWB pulses. The presented antenna operates in the UWB frequency range, i.e., 3.1–10.6 GHz. To improve the impedance matching of the antenna in presence of a coupling medium, and to protect the antenna from the effects of the coupling liquid, the antenna was covered by a dielectric material with properties similar to those of the substrate. As expected, the time-domain antenna performance shows that the fidelity factor decreases in the presence of human body. However, by using a coupling medium, the value of the fidelity factor is kept high (≈60%).

When dealing with planar antenna structures, the use of a substrate having high dielectric permittivity is considered to be the simplest solution to reduce the antenna size. As the $\epsilon_{r}$ of the dielectric substrate increases, wavelength decreases, and the size of the antenna reduces, allowing for a miniaturized design. For example, in [64,65], the RT6010 substrate with a dielectric constant of 10.2 was used. The AVA designed in [65], having dimensions of 50 mm × 50 mm, offers a 10-dB return loss bandwidth from 2.75 GHz to more than 11 GHz, while its gain varies between 3.5 and 9.4 dBi over the operating frequency range. In [66], a miniaturized conventional AVA design operating in the frequency range of 0.5–3 GHz was presented for a microwave imaging system. The proposed AVA was designed to work inside a coupling liquid having $\epsilon_{r}$ = 25 and a dielectric substrate with $\epsilon_{r}$ = 37 was used to reduce the antenna dimensions.

In [37], the authors designed compact AVAs to be used in a microwave imaging system for monitoring thermal ablation of liver tumors. In particular, the presented antennas were designed on three different substrates, namely Rogers RT/Duroid 6010LM ($\epsilon_{r}$ = 10.2), T-Ceram E-20 ($\epsilon_{r}$ = 20), and T-Ceram E-37 ($\epsilon_{r}$ = 37). Although the AVA designed on T-Ceram E-37 realized a compact size (42 mm × 50 mm), Rogers RT/Duroid 6010LM based AVA offered better impedance matching in the frequency range of 0.5–5 GHz. To excite the designed antennas in the presence of a coupling medium, a coaxial connector was used, and its pin was covered using an epoxy resin having $\epsilon_{r}$ = 4. In this way, it is possible to isolate the connector pin from the coupling medium, thus avoiding the connector impedance mismatch.

A comparison among different Vivaldi antennas performance is reported in Table 2. In the table, the dimensions of the antennas are reported both as absolute values and as values normalized to the wavelength. To this end, the wavelength corresponding to the central frequency of the antenna band was considered. From the table it can be noted that most Vivaldi antennas offer a FBW >110% and a high gain even though they are manufactured over high permittivity substrates to achieve compact size. The best performance, with respect to FBW, was achieved in [49,50,56,57] where the authors utilized low permittivity substrates, e.g., FR-4 ($\epsilon_{r}$ = 4.4). Low dielectric permittivity substrates are preferable as they reduce the levels of surface waves responsible for the degradation of antenna performance. In terms of normalized dimensions, the best performance with minimum dimensions was achieved in [66] where a high permittivity substrate was used.

### 4.2. Planar Monopole Antennas

Planar monopole antennas received a lot of attention from researchers because they are compact and lightweight. The basic structure of the planar monopole antenna is composed of a radiating structure either fed by a microstrip line or by a co-planar waveguide (CPW). The backside of the antenna consists of a full or partial ground plane. The full ground plane offers directional radiation characteristics, but at the expense of a narrow bandwidth. To enhance the impedance bandwidth of planar monopoles, a partial ground plane has been utilized by many researchers. The CPW feeding technique is used due to several advantages, e.g., low losses, co-planar nature, ease of fabrication, etc. Due to the above-mentioned advantages, many researchers proposed monopole design techniques for near-field microwave imaging applications. With these kinds of antennas, it is possible to achieve a FBW of 100%. The achievable gain is about 5 dBi. However, the gain of the actual monopole antenna with typical ground systems is around 2–3 dBi. Finally, the typical fidelity factor is about 75% [35].

#### 4.2.1. Techniques Employed to Enhance the Bandwidth of Planar Monopole Antennas

The operational bandwidth of a simple planar monopole antenna can be increased by applying suitable perturbations on the ground plane. In [67], two elliptical-shaped UWB planar antennas were designed for breast cancer detection. The top side of both antennas was formed by an elliptical-shaped patch radiator fed by a 50 Ω microstrip line, while the bottom side of the antennas consisted of a partial ground plane with two different rectangular slot configurations. In the first design, the rectangular slots were oriented at 45°, while in the second design the slots were oriented at 90°. Both antennas operate in the frequency range between 3–20 GHz (FBW = 147.82%).

**Figure 13:**
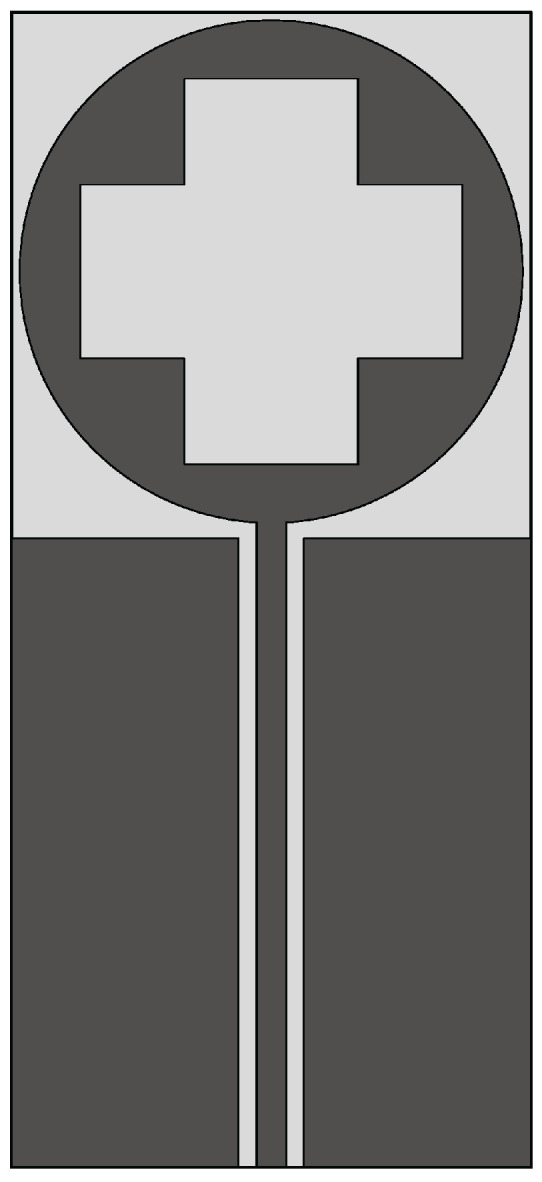
CPW-fed circular slotted planar monopole antenna for microwave imaging systems (redrawn from [69]). Dark gray metal face and light gray dielectric substrate.

In [68], a modified square monopole antenna design was presented for UWB microwave imaging systems. A pair of loop sleeves were designed within the ground plane, which allowed the antenna to operate in the range of 3.19 GHz to 11.03 GHz (FBW = 110.26%). To demonstrate the effectiveness of the antenna designed for a microwave imaging system, the SAR distribution in a water-filled phantom using six elements positioned in a circular configuration was evaluated, and it was observed that the specific absortion rate (SAR) distribution was uniform across the whole operating frequency range. Danjuma et al. [69] designed a CPW-fed quasi-cross slot-based circular monopole antenna, shown in Figure 13, for body-centric imaging applications. The antenna operates from 3 GHz to more than 11 GHz (FBW = 114.28%). To verify the antenna performance when placed close to a phantom, experiments were carried out and it was observed that the antenna maintains the UWB performance for different kinds of phantom tissues.

**Figure 14:**
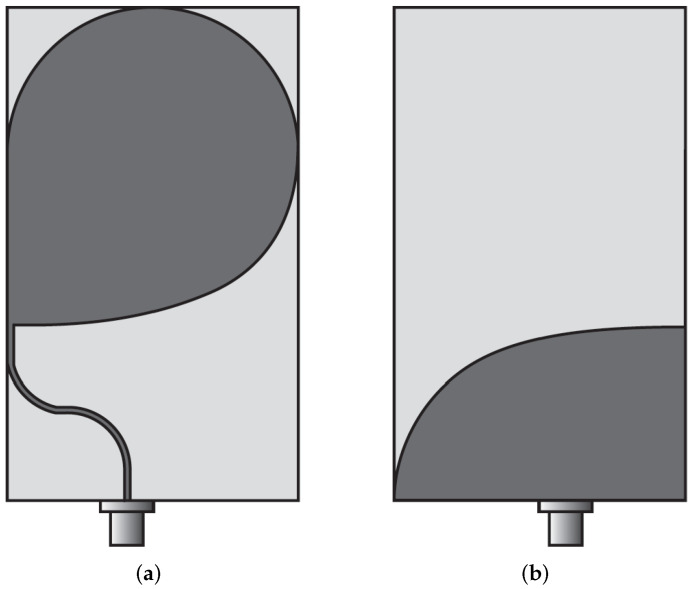
Half-heart shaped planar antenna to monitor cardiac activities (**a**) top metal (**b**) bottom metal (redrawn from [70]). Dark gray metal face and light gray dielectric substrate.

Pittella et al. [70] presented two half-heart shaped antenna designs to monitor cardiac activities (see Figure 14). The designed antennas had the same shapes, but they were printed on different dielectric substrates to achieve different antenna dimensions. One antenna is meant for wearable applications, while the other one is for non-wearable applications. The dielectric substrates utilized for the design are Rogers RO4003 with $\epsilon_{r1}$ = 3.38, $h_{1}$ = 0.508 mm, and Rogers RT6010 with $\epsilon_{r2}$ = 10.2, $h_{2}$ = 0.640 mm. The half-heart-like radiator was printed on the top side of the dielectric substrate. The antenna has a behavior similar to a Vivaldi antenna radiating along the horizontal direction (see Figure 14). The microstrip feed line was curved away from the edge of the structure to facilitate the connection with the SMA connector. Both antennas are well matched between 3.1 and 10.6 GHz (FBW = 110%).

**Figure 15:**
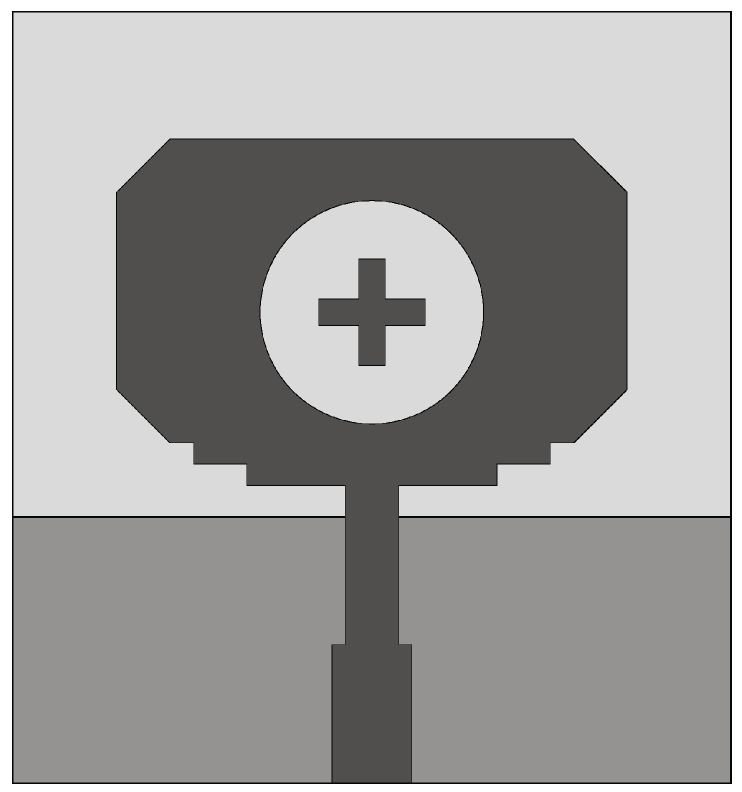
Design of a modified octagonal shape UWB monopole (redrawn from [72]). Dark gray top metal face, medium gray bottom metal face, and light gray dielectric substrate.

Mahmud et al. [71] designed a Hibiscus petal pattern-like patch antenna for microwave imaging systems. A tapered microstrip feed line and a partial trapezoid ground plane were used to achieve an impedance bandwidth in the frequency range of 3.04–11 GHz (FBW = 113.39%). The Hibiscus design exhibits a fidelity factor of ≈84% for face-to-face configuration and 92% for side-by-side configuration. In [72], a modified octagonal shaped UWB antenna fed by a microstrip and a step impedance transformer was designed for breast cancer detection (see Figure 15). The modified octagonal monopole, printed on the top side of the dielectric substrate, is excited by a 50 Ω microstrip feeding line. To improve the radiation performance of the antenna, the modified octagonal monopole was loaded with a circular slot including symmetrical metal cross (plus sign). The presented antenna configuration provides an impedance bandwidth of 12 GHz in the frequency range of 3–15 GHz (FBW = 133.33%).

**Figure 16:**
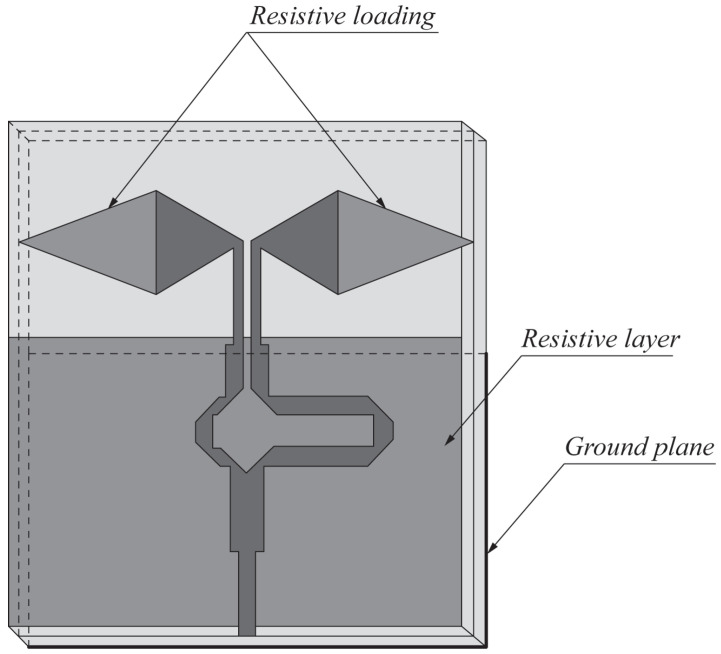
“Dark Eyes” planar antenna for near-field pulsed biological sensing (redrawn from [73]). Dark grey top metal face, medium grey resistive metal face, black bottom metal face, and light grey dielectric substrate.

In [73], a microstrip-fed “Dark Eyes” antenna was designed for near-field pulsed biological sensing. The designed antenna was printed on Rogers RT/Duroid 6010 laminate coated with resistive materials. This laminate presents dielectric properties close to those of fat ($\epsilon_{r}$ = 9 @ 6 GHz). The presented antenna is a dual-layer planar structure, with the radiating element and the microstrip feed embedded between two dielectric layers as shown in Figure 16 [73]. A resistive parasitic cover designed by Ohmega Technologies (http://www.ohmega.com, (accessed on 19 April 2022)) was introduced over the top dielectric layer to reduce the radiations generated from the microstrip feed line, while the ground plane was designed on the outer surface of the bottom dielectric layer as shown in Figure 16. Simulation results showed that the antenna operates from 2.7 GHz to 9.7 GHz (FBW = 113%).

**Figure 17:**
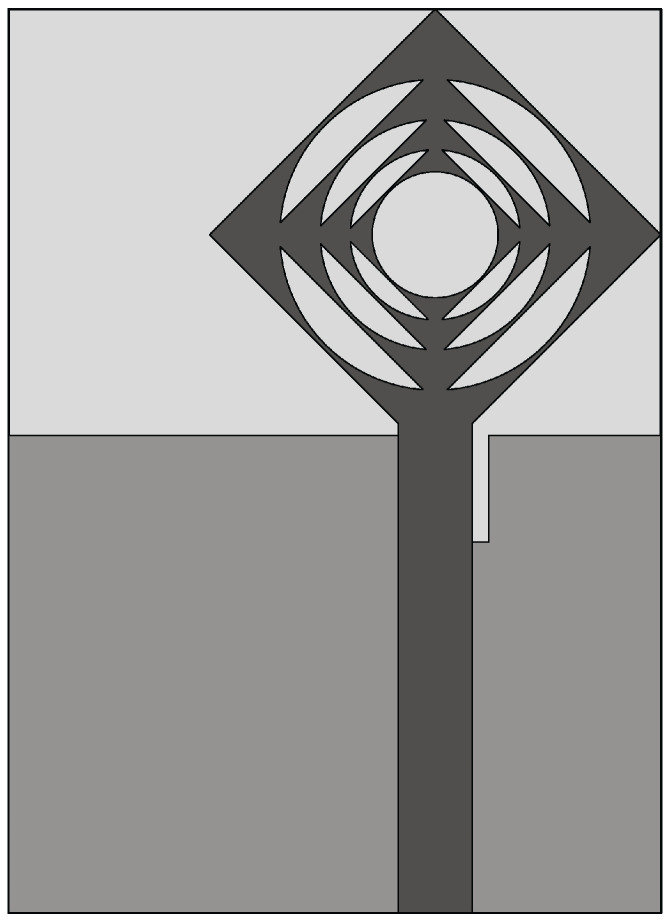
Geometry of a planar fractal antenna for UWB imaging applications (redrawn from [76]). Dark gray top metal face, medium gray bottom metal face, and light gray dielectric substrate.

Lee et al. [74] presented a UWB resistively-loaded dipole antenna for radar-based monitoring of skull healing using cranial surgery phantom models. They found that the designed resistive dipole (RD) has less internal reflection compared to other UWB antennas. To achieve this requirement, chip resistors were placed on both arms of the dipole using the Wu-King profile [75]. They also showed that the presented RD is suitable for short pulse radiating applications in the near-field zone. In particular, it was observed that according to −15 dB impedance bandwidth criteria, the antenna operates from 1 GHz to 10 GHz (FBW = 163.63%). In [76], a square fractal monopole antenna with a slotted partial ground plane, shown in Figure 17, was presented for imaging applications. Measured results demonstrate that the fractal antenna offered FBW of 117.88% from 3.1 GHz to more than 12 GHz.

#### 4.2.2. Techniques Useful to Enhance Planar Monopole Radiation Characteristics

**Figure 18:**
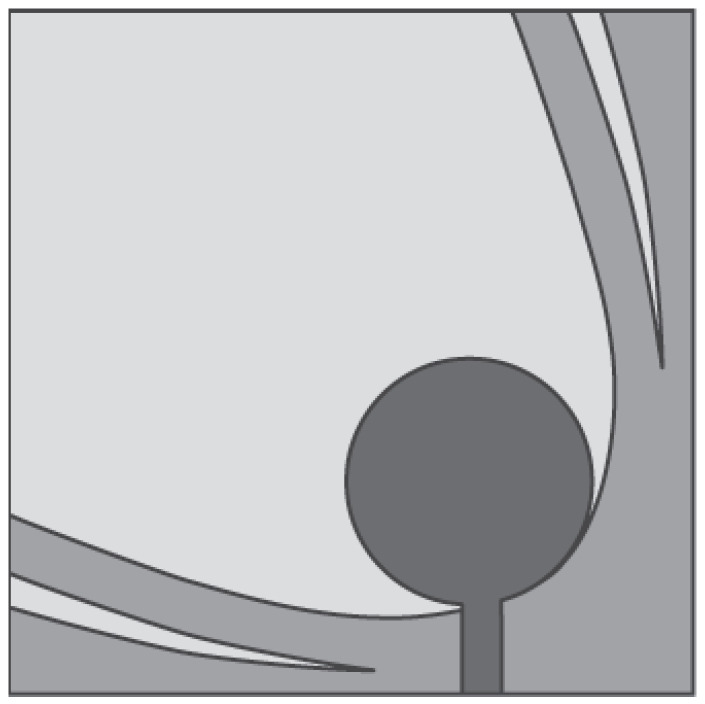
A modified parabolic-shaped ground plane-based directional circular antenna (redrawn from [77]). Dark gray top metal face, medium gray bottom metal face, and light gray dielectric substrate.

In [77], a directional circular monopole antenna was designed with improved directivity and radiation characteristics. For this purpose, a parabolic-shaped ground plane was utilized. The parabolic curve was rotated 45° to extend its axis in the direction of the substrate diagonal, as shown in Figure 18. Furthermore, for improved radiation characteristics, two parabolic slots were realized along the corners of the ground plane. Measured results showed that the antenna is matched from 4 GHz to 9 GHz, while the gain varies in the range of 7–10.5 dBi. Moreover, the −3 dB aperture of the radiation pattern varies between 22–54° in the entire operating range due to the presence of the parabolic-shaped ground plane, which acts as an antenna reflector. The presence of slots etched from the ground plane determines an increase in the gain [67]. For 45° oriented slots, the gain ranges between 7.4 dBi to 8.81 dBi, while for 90° oriented slots, the gain varies in the range of 7.15–9.08 dBi from 3 GHz to 20 GHz. The shaping of the patch is also able to produce an increase in the fidelity factor.

#### 4.2.3. Techniques Utilized to Reduce Planar Monopole Antenna Dimensions

**Figure 19:**
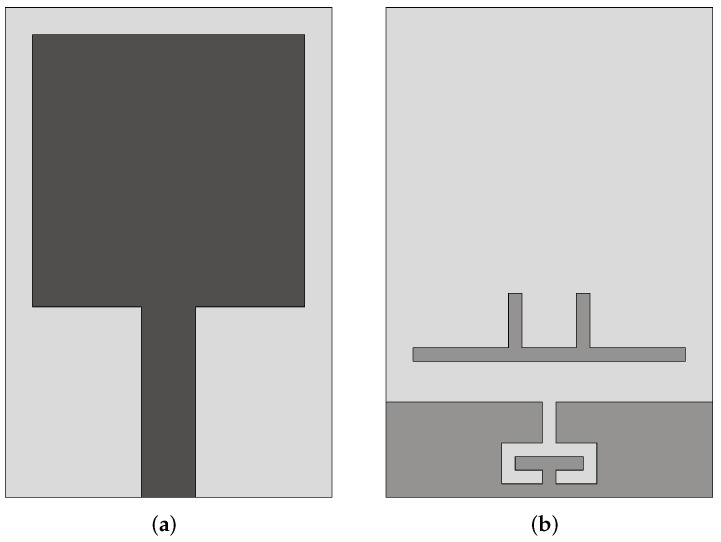
Geometry of the modified ground plane monopole antenna (**a**) front view and (**b**) back view (redrawn from [79]). Dark gray top metal face, medium gray bottom metal face, and light gray dielectric substrate.

Miniaturized antennas can be realized by making slots in the ground plane or by using parasitic elements. For example, in [78] a modified square planar antenna having dimensions 10 cm × 10 cm was designed and presented for UWB medical applications. By modifying the square patch and also utilizing inverted U-shaped parasitic elements, an enhanced impedance bandwidth was demonstrated from 3.8 GHz to more than 9 GHz. In addition, the proposed antenna offered a fidelity factor of about 90%. In [79], an open circuit T-shaped slot was etched from the ground plane of a square monopole antenna to achieve an overall size of 12 mm × 18 mm. In addition, a π-shaped parasitic element was placed behind the microstrip line (see Figure 19). By utilizing a T-shaped slot and π-shaped parasitic element, the bandwidth of the antenna extends from 3.12–10.45 GHz to 2.91–14.72 GHz (FBW = 134%).

The technique presented in [79] was also used by Ojaroudi and Ghadimi [80] to design a multi-resonance monopole antenna for microwave imaging systems. In their design, a rotated E-shaped slot was etched in the ground plane, and an E-shaped parasitic structure was designed behind the microstrip feed line. By utilizing this configuration, the upper-frequency limit of the antenna extended from 10.3 GHz to 15 GHz. The overall dimensions were 12 mm × 18 mm and FBW of designed antenna was >135% since the band was from 2.9 GHz to 15 GHz. The same authors designed square monopole antennas in [81,82]. In particular, in [81], L-shaped slots were inserted in the ground plane of a square monopole, while in [82], E-shaped slots and T-shaped slits were introduced in the ground plane. For the above-presented antenna designs, the fidelity factors for both face-to-face and side-by-side configurations are 91% and 84%, respectively. In [83], three T-shaped slots were etched on the ground plane. In particular, one slot was placed between two rotated T-shaped slots. This design operates in the frequency range of 2.96–15.8 GHz with a maximum measured gain of 6.1 dBi.

**Figure 20:**
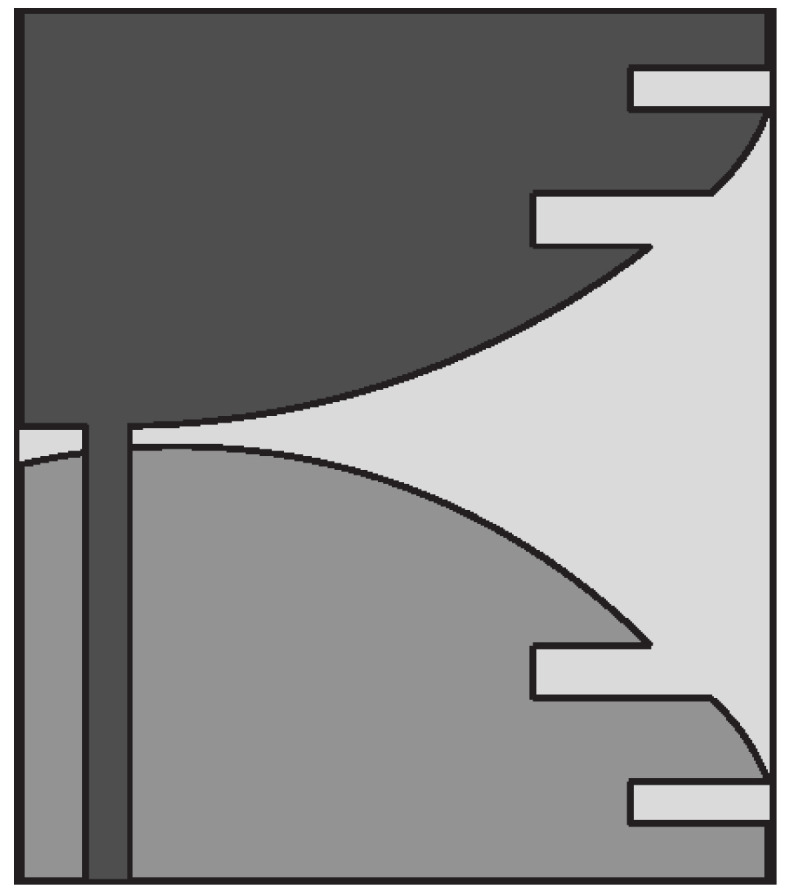
Design layout of a compact corrugated planar antenna (redrawn from [84]). Dark gray top metal face, medium gray bottom metal face, and light gray dielectric substrate.

In [84], a very compact size (9 mm × 10 mm) UWB antenna was designed and presented for microwave imaging systems. Both the monopole, printed on the upper face of the substrate, and the ground plane at the bottom face have the shape of a quarter of an ellipse. To improve the antenna’s performance, pairs of symmetrical slots were etched from the radiator and the ground plane (see Figure 20), which increased the current path near the termination of the antenna radiating arms. The results showed that the measured 10 dB return loss bandwidth extends from 2.1 GHz to more than 11 GHz. Furthermore, the time domain analysis of the antenna shows negligible distortion in the received pulse, which makes it suitable for medical imaging systems.

**Figure 21:**
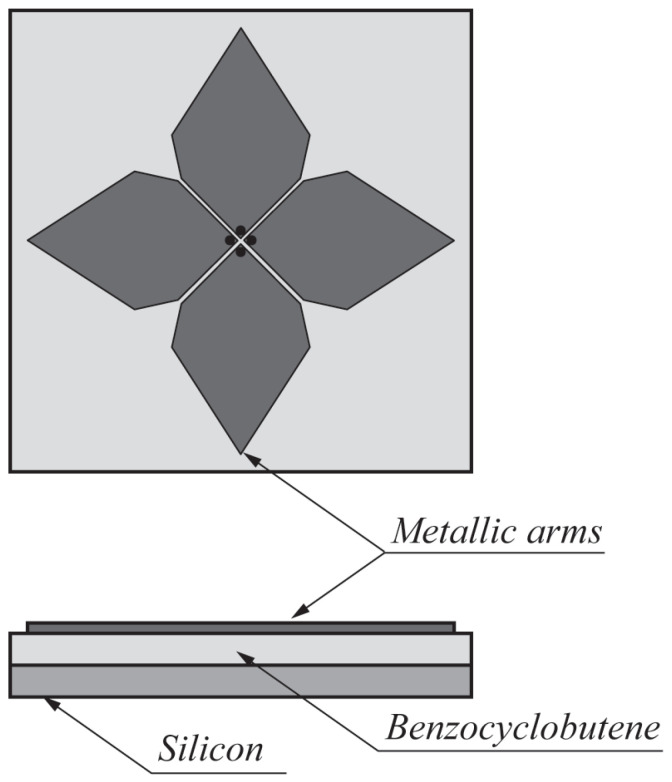
Configuration of the fourtear antenna structure (redrawn from [85]). Dark gray top metal face, medium gray bottom dielectric substrate, and light gray center dielectric substrate.

**Figure 22:**
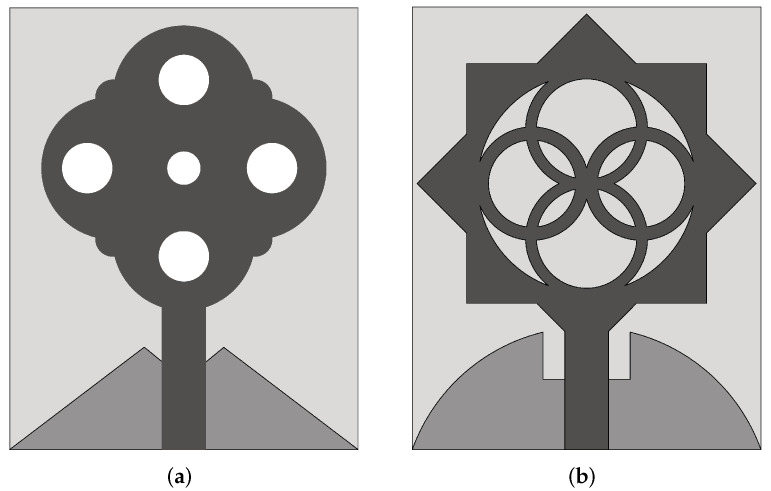
Miniaturized fractal antenna designs (**a**) Sierpinski gasket (**b**) rings inscribed hexagonal patch (redrawn from [86]). Dark gray top metal face, medium gray bottom metal face, and light gray dielectric substrate.

A planar antenna capable of providing dual linear polarization was designed in [85] for the detection of breast tumors (see Figure 21). The radiation element was based on a fourtear structure. Two substrate layers of silicon ($\epsilon_{r}$ = 11.7) and benzocyclobutene (BCB) having $\epsilon_{r}$ = 2.65 were used for the design. The overall size of the antenna was 15 mm × 15 mm. By utilizing identical arms, the antenna offered dual linear polarization in the operating range of 5–10 GHz. The same antenna was embedded in oil, to be used as a coupling liquid ($\epsilon_{r}$ = 3) with the breast tissue. In this configuration, the antenna provides an impedance bandwidth of 4 GHz in the frequency range of 3.8–7.8 GHz. Lasemi and Atlasbaf [86] presented miniaturized UWB fractal monopole antennas for breast cancer detection. One antenna design was based on the Sierpinski gasket configuration (see Figure 22a) having dimensions 20 mm × 28 mm, and the other one was a rings-inscribed hexagonal-shaped fractal antenna with dimensions of 16 mm × 22 mm (see Figure 22b). Measurement results showed that the designed antennas operate in the frequency range of 2.95–12 GHz (FBW = 121%).

**Figure 23:**
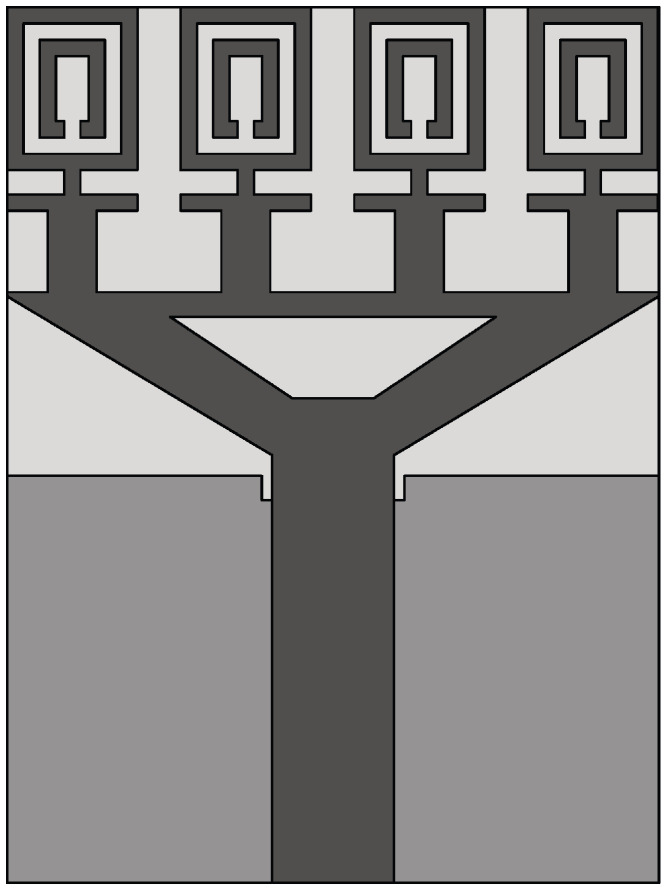
Negative index metamaterial-based planar monopole antenna (redrawn from [87]). Dark gray top metal face, medium gray bottom metal face, and light gray dielectric substrate.

In [87], a compact negative index metamaterial-based UWB planar antenna design was presented for microwave imaging applications. Four left-handed (LH) metamaterial (MTM) unit cells were placed on the upper edge of a triangular monopole equipped with a slot (see Figure 23). Each LH-MTM unit cell consisted of a modified split-ring resonator (SRR) and a capacitance-loaded strip to obtain both negative permittivity and permeability, which ensured a stable negative refractive index. Through this configuration, the achieved antenna dimensions were 16 mm × 21 mm. Measured results illustrate that the antenna offered 114.5% FBW in the frequency range of 3.4–12.5 GHz with a maximum gain of 5.16 dBi at 10.15 GHz. To validate the time-domain performance of the proposed antenna, two identical antennas were placed 30cm apart from each other. In this condition, the signal received by the second antenna showed limited distortion. They also observed a fidelity factor of 91% and 83% for face-to-face and side-by-side configurations, respectively.

The authors in [88] utilized the same technique as presented in [87] to design a compact metamaterial-based UWB planar antenna. In particular, the top side of the antenna consists of a trapezoid-like patch radiator fed using a stepped tapered microstrip feed line, while the bottom side is formed by a partial ground plane. Furthermore, the radiating patch consists of four metamaterial unit cells, which help to achieve a reduced antenna size (10.2 mm × 15.5 mm). The presented antenna was able to operate in the frequency range between 4.23 GHz to more than 14 GHz (FBW = 107%), where the gain value varies between 2 dBi to 5.17 dBi. In addition, the antenna fidelity factor is >95%, which is higher than the design presented in [87]. In [89], a full ground plane-based UWB wearable textile antenna having dimensions 50 mm × 60 mm was designed for breast cancer detection. To reduce the size, a substrate with a high dielectric constant was selected. The presented antenna was based on photonic bandgap (PBG) structures and substrate integrated waveguide (SIW) technology. The antenna was fed using a grounded CPW (GCPW) through a SIW transition. The designed antenna had a FBW of 120% from 7 GHz to 28 GHz, maximum gain of 10.1 dBi, and maximum radiation efficiency of 94.9%.

A comparison among the performances of planar monopole antennas designed for biomedical applications is provided in Table 3. In the table, the dimensions of the antennas are reported both as absolute values and as values normalized to the wavelength. To this end, the wavelength corresponding to the central frequency of the antenna band was considered. From the table it can be noted that most of the antennas are realized with low permittivity substrates to achieve compact size. In addition, most of the planar monopole antennas provide FBW > 110%, which is the basic requirement for UWB operation. The best performance in terms of FBW was achieved in [67,74] where the authors utilized low permittivity dielectric substrates. In terms of normalized dimensions, the best performance was achieved in [84] with the use of high permittivity substrate.

### 4.3. Planar Slot Antennas

**Figure 24:**
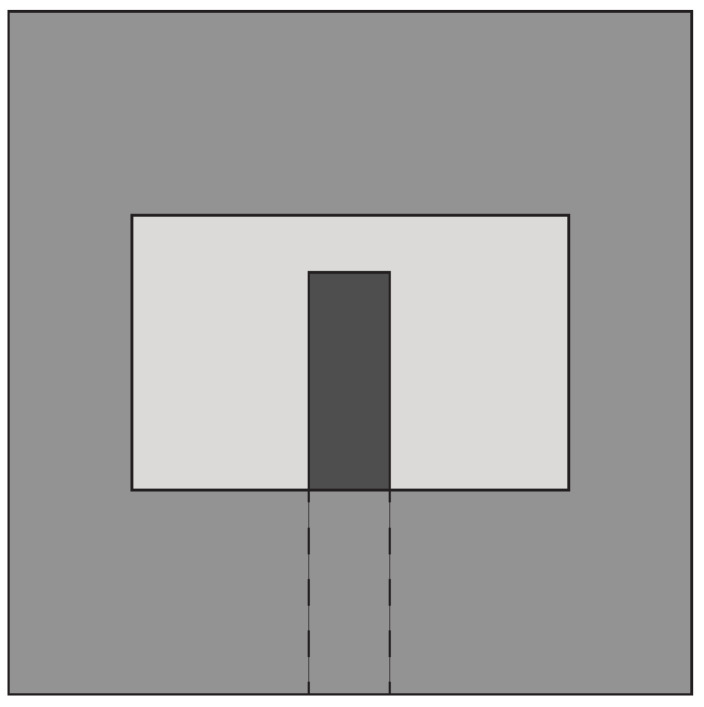
Geometry of a planar slot antenna (redrawn from [93]). Dark gray top metal face, medium gray bottom metal face, and light gray dielectric substrate.

The conventional geometry of a planar slot antenna consists of a narrow slot etched from the ground plane, while a microstrip feed line is printed on the other side of the substrate, as shown in Figure 24. By utilizing this configuration, a good impedance matching can be achieved, and FBW of about 20% can be obtained [90]. As the width of the slot increases, the radiation resistance ($R_{r}$) of the slot also increases, which in turn reduces the impedance bandwidth of the antenna [91]. A wide impedance bandwidth can be achieved by properly terminating the open end of the feed line. Furthermore, center-feed [90] or offset-feed [92] excitation systems are used to excite the radiating slot. The impedance bandwidth of the center-fed slot antenna is lower compared to the offset case because the center-fed has a larger value of radiation impedance.

To improve the radiation characteristics, the antenna can be either backed by a reflector or a cavity. However, the use of these structures leads to a bulky design and adds further design complexities. Different slot antenna designs have been presented in the literature [93,94,95]. Some of these antennas are meant for near-field microwave imaging applications, details of which are provided in the upcoming sections.

#### 4.3.1. Techniques Employed to Enhance Slot Antennas Bandwidth

**Figure 25:**
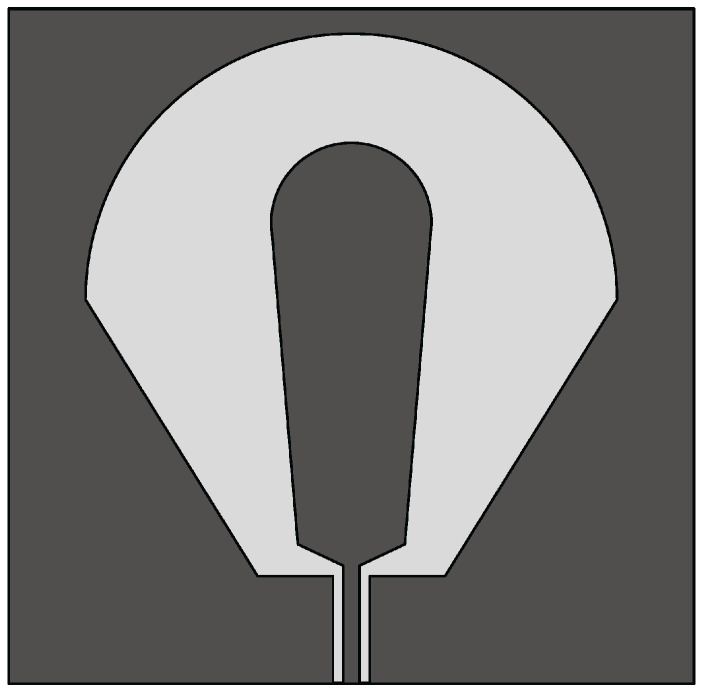
Schematic of a CPW-fed tapered arc slot antenna (redrawn from [97]). Dark gray metal face and light gray dielectric substrate.

A CPW feeding technique is generally employed to feed slot antennas because it allows to directly excite the radiating slot, making the structure more compact and easier to realize. Tavassolian et al. [96] proposed a flexible uni-planar CPW-fed UWB slot antenna configuration for tumor detection. The antenna consists of a CPW-feed elliptical slot and an irregular U-shaped stub realized on a flexible liquid crystal polymer (LCP) having $\epsilon_{r}$ = 3. The antenna works from 2 GHz to 12 GHz (FBW = 142.85%). Hossain et al. [97] made a CPW-fed wideband slot antenna excited by a tapered monopole. To increase the operating bandwidth, a tapered arc slot was adopted (see Figure 25). A tuning stub, approximately one-third of the slot size, was positioned inside the radiating slot thereby achieving an impedance bandwidth of 9.69 GHz in the 2.89–12.58 GHz frequency range (FBW = 125%). The performance of the antenna was tested using different coupling mediums such as margarine, soybean oil, and Vaseline, and a FBW = 100% was observed.

See et al. [98] designed a CPW-fed slot antenna and evaluated the proximity effects of the human body. The antenna was formed by using a monopole-like slot and a CPW fork-shaped feeding structure printed on the same side of an FR-4 substrate. The slot and the fork-shaped feeding structure were positioned symmetrically with respect to the slot geometry. The authors demonstrated that the use of this feeding structure can provide effective coupling between the slot and the feeding structure, thus achieving a broader impedance bandwidth. In addition, they noticed that the operating bandwidth can be controlled by changing the width of the slot, while the lowest and upper edge operating frequencies can be adjusted by tuning the length of the horizontal ground strips and horizontal section of the fork-shaped feeding structure. The designed antenna demonstrated good impedance matching in the 3.1–10.6 GHz band. When the antenna was placed at a distance of 1 mm from the human body, the reflection coefficient drops to −5 dB. A similar design was reported in [99] where a tapered slot with a fork-shaped feeding structure was used to excite the antenna, obtaining an impedance bandwidth of 6 GHz from 2 GHz to 8 GHz (FBW = 120%). The performance of the antenna was mantained when the antenna was measured facing a coupling medium whose electrical properties were similar to those of the fatty tissue.

#### 4.3.2. Techniques Used to Design Miniaturized Slot Antennas

**Figure 26:**
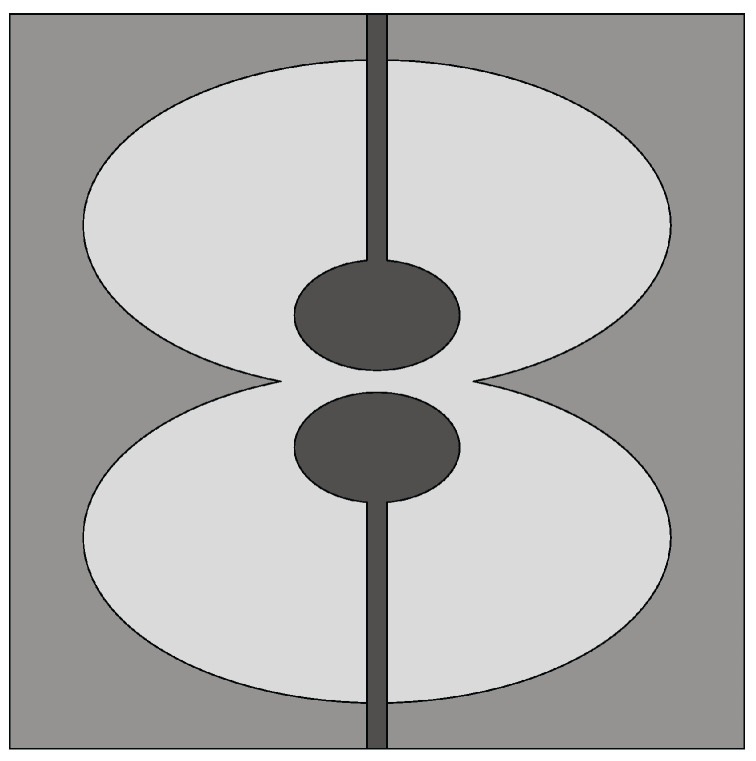
Layout of the double-elliptical slot antenna (redrawn from [100]). Dark gray top metal face, medium gray bottom metal face, and light gray dielectric substrate.

**Figure 27:**
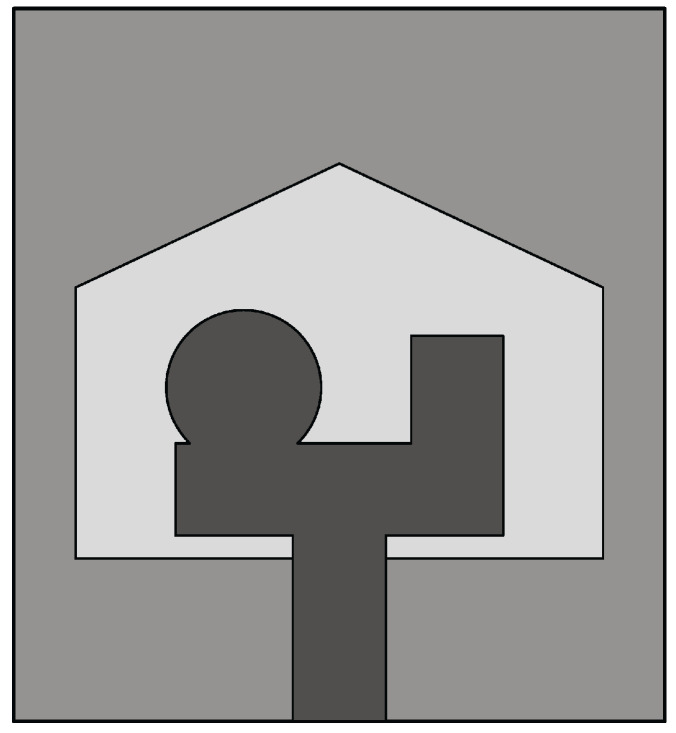
Design of the compact hut-shaped slot antenna (redrawn from [102]). Dark gray top metal face, medium gray bottom metal face, and light gray dielectric substrate.

In [100], a double elliptical slot antenna design with reduced dimensions (25 mm × 36 mm) was presented for biomedical applications. Two elliptical-shaped radiators were designed on the top side of the substrate, while the double elliptical slot-based ground was designed on the bottom side, as shown in Figure 26. The elliptical radiators were differentially fed by two tapered microstrip lines having a phase difference of 180°. The double elliptical slot on the ground plane increases the path of the magnetic equivalent currents, thus achieving enhanced impedance bandwidth. A differential feeding network was also integrated into the antenna to create a compact structure capable of operating in the 1–9 GHz frequency range with a constant gain (>8 dBi) from 2 GHz to 8 GHz. These performances were maintained when the antenna was considered in the presence of a coupling material. The same authors designed a stepped slot-based dual-polarized elliptical antenna in [101] for medical diagnostic applications. The stepped slot allows bandwidth enhancement and antenna size reduction (50 mm × 50 mm). Islam et al. [102] designed a compact slot antenna of dimensions 23 mm × 21 mm for breast tumor detection. Two monopoles, one rectangular and one circular, excite a house-shaped slot (see Figure 27). The antenna has an average gain of about 4.5 dBi in the 3.1–12 GHz band, and presents a fidelity factor of 87% and 91% when face-to-face and side-by-side configurations are considered.

In Table 4, a comparison among planar slot antennas, which are used for near-field microwave imaging is reported. In the table, the dimensions of the antennas are reported both as absolute values and as values normalized to the wavelength. To this end, the wavelength corresponding to the central frequency of the antenna band was considered. All the designs in the table show FBW ≥ 110%. In terms of FBW, the best performance was achieved in [96]. Minimum antenna dimensions with respect to the wavelength were achieved in [99] where the authors used high permittivity substrate.

### 4.4. Bowtie Antennas

The bowtie antenna is a planar version of the biconical antenna that can be easily realized on a planar substrate. It consists of two triangular arms etched on a printed circuit board and fed at the arms’ apex [103]. As the currents are abruptly terminated at the ends of the bowtie fins, the antenna has some bandwidth limitations compared to 3-D biconical [104] and discone antennas [105]. Still, the bowtie antenna shows good impedance behavior across a wide band (FBW = 37%) [106].

#### 4.4.1. Techniques Useful to Enhance Bandwidth of Bowtie Antennas

The bandwidth of the conventional planar bowtie antenna can be increased to 40% by realizing a bowtie slot antenna [107]. By further introducing tapering to the metal stubs at the center of the bowtie slot antenna, an even wider impedance bandwidth (FBW = 70.96%) can be achieved [107]. Due to the tapered shape, the VSWR of the bowtie does not degrade significantly as the frequency increases. Furthermore, by optimizing the stub parameters it is possible to create a shift in the lower resonant frequency, and generate an additional resonance process in the higher frequency band, thereby resulting in an improved antenna bandwidth.

In [108], a modified slot-line bowtie hybrid antenna, immersed in a coupling medium having $\epsilon_{r}$ = 9.5, was presented for cancer detection applications. A strip-slot UWB balun was designed for antenna feeding. The antenna consisted of shaped slots having Vivaldi, linear, and elliptical sections. The shaping of the slot allows control over internal reflections. Furthermore, bowtie plates were attached to the slot to provide control over antenna beamwidth. The bowtie plates were encased in epoxy for improved impedance matching and structural support. The results showed that, according to 10 dB bandwidth criteria, the antenna operates from 2.5 GHz to 9 GHz (FBW = 113%).

#### 4.4.2. Techniques Employed to Enhance Radiation Characteristics of Bowtie Antennas

**Figure 28:**
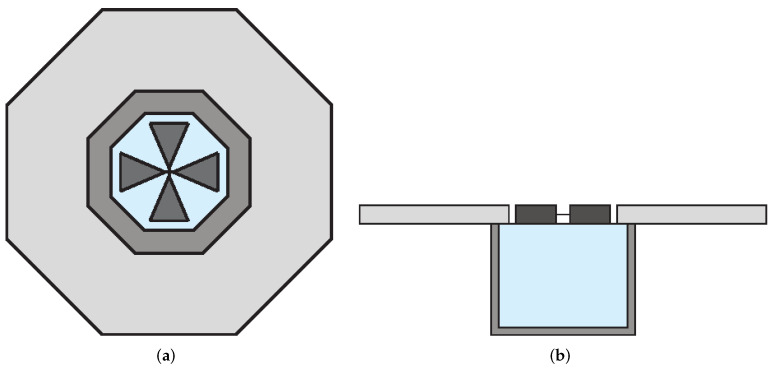
(**a**) Top view and (**b**) side view of the crossed bowtie antenna (redrawn from [112]). Dark gray top metal face, medium gray metal flange, light blue cavity face, and light gray dielectric substrate.

To enhance the radiation characteristics of the bowtie antenna, a cavity-backed structure can be utilized [109]. This design technique has the advantage of obtaining a unidirectional radiation pattern with high gain, low sidelobes, and backlobes [110,111]. In [112], a compact bowtie antenna design was presented to detect cross-polarized reflections generated from breast tumors. The presented antenna configuration consisted of two crossed bowtie elements, an octagonal cavity behind the bowtie elements, and a metal flange attached to the cavity, as shown in Figure 28. Both bowties had a flare angle of 45°, and they were meant to operate at the center frequency of 3 GHz. The presence of the metallic flange forces surface waves into the dielectric, rather than allowing propagation along with the interface. Additionally, to match the antenna to the breast, a homogeneous medium with properties close to those of fatty tissue ($\epsilon_{r}$ = 9) surrounds the antenna.

**Figure 29:**
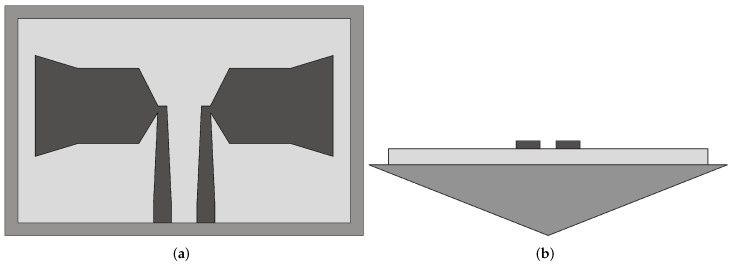
Design layout of the bowtie antenna (**a**) geometry of bowtie (**b**) pyramidal reflector (redrawn from [113]). Dark gray top metal face, medium gray cavity face, and light gray dielectric substrate.

Kwag et al. [113] designed a novel bowtie antenna with a pyramidal shape for UWB radar imaging applications. The radiating element consists of a pair of horn-shaped tapered bowtie arms, while a tapered slot line is placed between the bowties, which are designed for better impedance matching (see Figure 29a). To achieve directional radiation properties, a horn-shaped pyramidal reflector is placed to the back of the bowtie arm, as shown in Figure 29b. For this antenna, the authors observed an impedance bandwidth of 8.64 GHz from 3.06 GHz to 11.7 GHz with a peak gain of 8.5 dBi.

To minimize the late ringing time of the bowtie antenna, which is caused by surface current reflections, resistive loading may be used [114,115]. In [116], a resistively loaded bowtie antenna was presented for cancer detection applications. The presented antenna was based on a wire bowtie structure. For size reduction and reduced surface current reflections, resistors of different values were placed on the radiating elements. These elements consist of three wires, and the flare angle between the wires is set to be 38°. Each wire had six resistors having values of 109 Ω, 17 Ω, 11 Ω, 113 Ω, 120 Ω, and 219 Ω. To match the electrical properties of human tissues at microwave frequencies, the antenna was immersed in a vegetable oil having $\epsilon_{r}$ = 9 and σ = 0.4 S/m. The results demonstrate that the resistive bowtie offered a UWB frequency response from 3 GHz to more than 10 GHz. The measured radiation efficiency of the bowtie antenna in free space was between 18% and 26% across the operating frequency band.

#### 4.4.3. Techniques Useful to Design Miniaturized Bowtie Antennas

**Figure 30:**
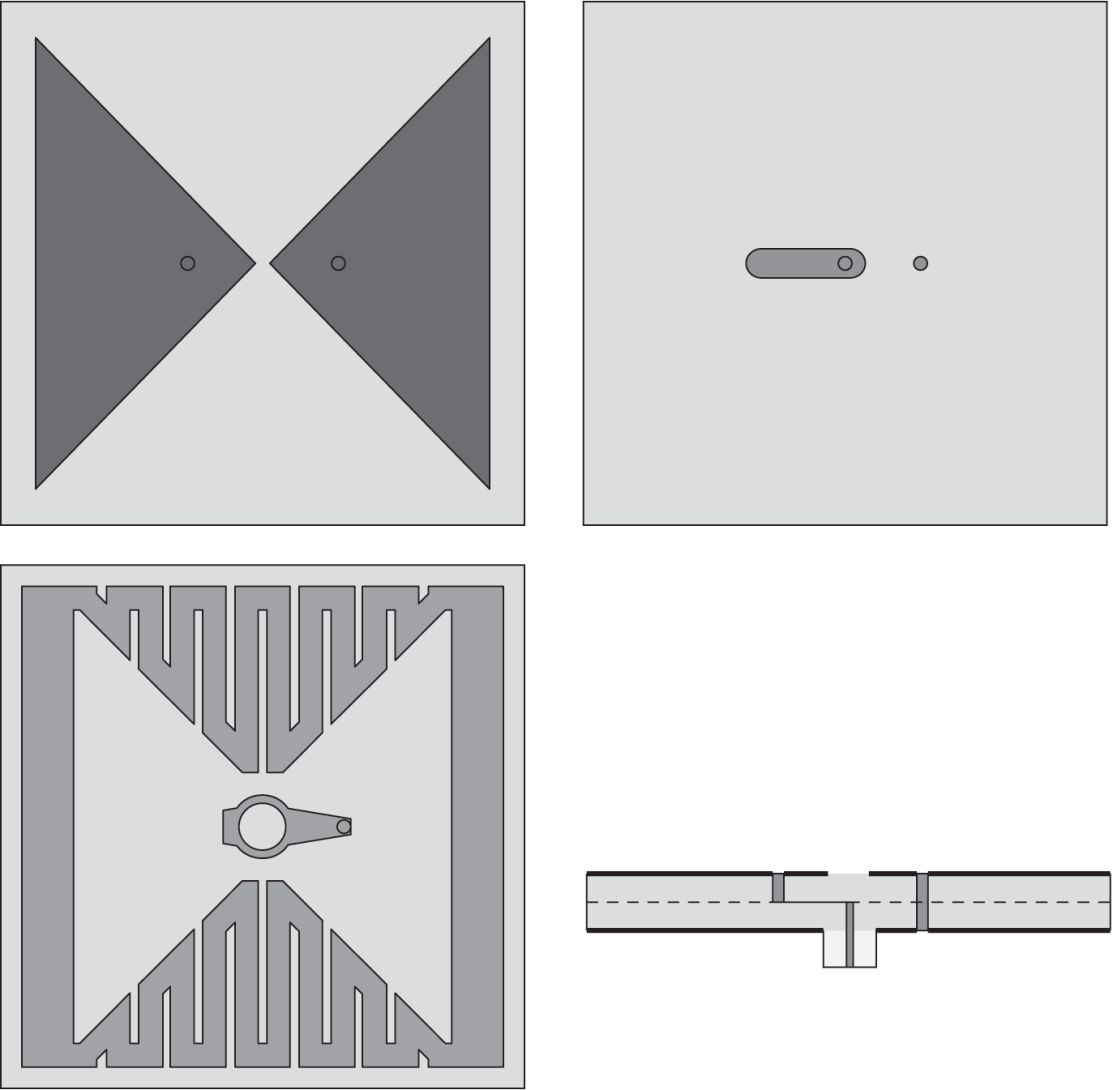
Design of the double-layer bowtie antenna (redrawn from [117]). Dark gray top metal face, medium gray center metal face, and light gray dielectric substrate.

The reduction in the bowtie antenna size can be done by introducing meandered lines within a double-layer structure. In [117], the bowtie antenna was matched to the human body to allow more energy to be radiated inside the body. This design showed a small size of 30 mm × 30 mm. The size was reduced by using a dual-layer bowtie antenna with a folded structure and meandered microstrip lines designed on the backside of the antenna (see Figure 30). The designed antenna operates from 0.5 GHz to 2 GHz. The same technique was utilized by [118]. In this design, the arms of the bowtie were connected to the meandered structure designed on the backside of the antenna, and the antenna was fed by a coaxial cable. The meandered connection was used to increase the path of current and thereby allow minimization of the antenna (22 mm × 30 mm). The meandered strips also enhanced the bandwidth of the antenna that operates in the frequency range of 0.85–3.5 GHz.

Table 5 presents a comparison among the bowtie antennas, which are described above. In the table, the dimensions of the antennas are reported both as absolute values and as values normalized to the wavelength. To this end, the wavelength corresponding to the central frequency of the antenna band was considered. The best performance, with respect to FBW, was achieved in [118]. Minimum dimensions with respect to the wavelength were obtained for the bowtie design presented in [117], thanks to the use of a high permittivity dielectric substrate. Nevertheless, FBW is close to the maximum achieved in the literature (120 vs. 121.83), as is the gain (7.5 vs. 8.5 dBi).

### 4.5. Horn Antennas

Horn antennas are widely used as a feed element for reflector antennas in many applications such as in satellite tracking systems, radio astronomy, radar, radio link wireless communication, etc [119]. In addition, it is employed in phased arrays and as a standard antenna for measuring the gain of other antennas. A horn can take different forms. The most commonly used are those adopted in E- and H-plane sectoral horn configurations, as well as those employed in pyramidal and conical shapes. The horn is nothing more than a hollow pipe of different cross-sections that is tapered toward a wider opening. So, to limit reflection effects, the performance of the horn can be handled by utilizing different types of tapering.

The total field radiated by a conventional horn is a combination of the direct field and those arising from edge diffraction occurring at the antenna opening, whose levels can be evaluated using diffraction techniques [120,121,122]. The edge diffractions, particularly those that occur at edges where the electric field is normal, influence the antenna pattern structure, especially in the back lobe region. The diffractions produce undesirable radiation which affects both the main and side lobe levels of the antenna radiation pattern.

One of the advantages of utilizing horn antennas is that they have no resonant elements, so it can operate over a wide range of frequencies, exhibiting antenna gains of the order of 10–20 dBi or higher. Due to these characteristics, horn antennas are widely utilized in microwave and millimeter-wave (mm-wave) applications. Some of the horn antennas presented in the literature for microwave imaging applications are discussed below.

#### 4.5.1. Techniques Employed to Enhance Horn Antenna Bandwidth

**Figure 31:**
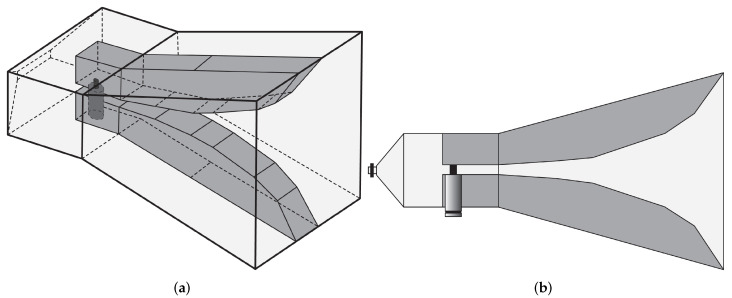
Geometry of the double-ridged horn antenna (**a**) 3-D view (**b**) side view (redrawn from [124]). Medium grey ridges and light grey outer metal.

Ridges can be applied to the horn antenna to enhance the operational bandwidth by extending the lower cut-off frequency of the incoming waveguide. In [123], a TEM horn antenna design was presented for near-field microwave imaging applications. A microstrip-to-parallel strip balun was also developed to properly match the coaxial connector impedance to that of the antenna. Numerical simulations and experimental results showed that the VSWR is lower than 2 in the frequency range of 2–12 GHz (FBW = 142.85%). Latif et al. [124] presented a dual-ridge horn antenna design for radar imaging applications. The impedance bandwidth of the antenna was increased by using ridges in the waveguide and flared sections as shown in Figure 31. This antenna exhibits an impedance bandwidth of 3.75 GHz between 1.54–5.29 GHz (FBW = 110%), based on a −7 dB criterion, and a peak gain of 7.8 dBi, when the antenna is immersed in coupling medium.

**Figure 32:**
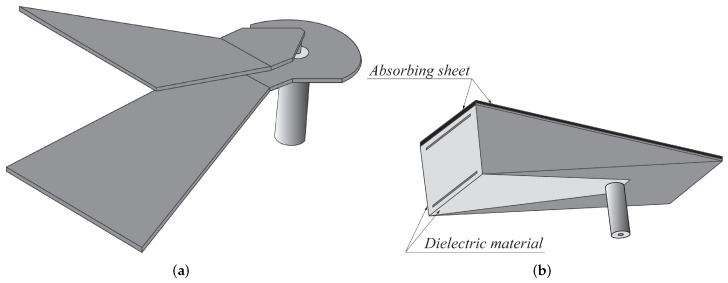
(**a**) 3D view of flared TEM horn antenna (**b**) The horn enclosed in a dielectric medium (redrawn from [125,126]). Dark gray metal face and light gray dielectric medium.

In [125,126], a transverse electromagnetic (TEM) horn was designed to detect breast tumor. It consists of two metal plates having a linear tapered structure connected to a coaxial cable by means of a balun (see Figure 32a) used to ensure impedance matching. The designed horn antenna was filled with a solid dielectric medium, shown in Figure 32b, having $\epsilon_{r}$ = 10 and tanδ = 0.01 so that it can be utilized for breast tumor measurements without a coupling liquid. In addition, copper sheets were placed on all outer surfaces of the horn except the front aperture, and a microwave absorbing sheet was placed on the top surface to reduce external surface currents responsible for the formation of lateral lobes and back radiation. The results showed that the proposed horn antenna operates from 3 GHz to 11 GHz (FBW = 114.28%).

#### 4.5.2. Techniques Used to Design Miniaturized Horn Antennas

In the previous sections, it was discussed that the use of high dielectric permittivity materials reduce antenna size. In [127], a ceramic material-based dual-ridge horn antenna was designed for biomedical UWB radar applications. By utilizing a ceramic material having $\epsilon_{r}$ = 70, a horn antenna with an overall aperture size of 16 mm × 11 mm was realized. The presented horn exhibits a gain of about 8 ± 3 dBi in the frequency range of 1.5–5 GHz. Although the design was compact, the use of high dielectric constant material affects the input impedance of the antenna reducing it to 15 Ω. To overcome this problem, the antenna was integrated with a UWB amplifier, which provided matching to the low input impedance of the antenna.

A comparative analysis among the above presented horn antennas is given in Table 6. In the table, the dimensions of the antennas are reported both as absolute values and as values normalized to the wavelength. To this end, the wavelength corresponding to the central frequency of the antenna band was considered. In terms of FBW, the best performance was achieved in [123]. On the other hand, in terms of normalized dimensions, the best performance was achieved in [127].

### 4.6. Dielectric Resonator Antennas

Dielectric resonator antennas (DRAs) are considered a valid alternative to traditional radiating elements (e.g., 3D monopoles, printed antennas, etc.), especially in high frequency applications. One of the main reasons for using DRAs is that they do not suffer from conduction losses and are characterized by high radiation efficiency when properly excited. Moreover, being 3D structures, they introduce further degrees of freedom to the design, which when suitably exploited allow to obtain radiative performances not achievable with other types of manufacturing technologies [128]. Generally, DRAs are realized by using ceramic materials of high permittivity and high quality factor (greater than 1000). Since high dielectric permittivity make DRAs narrow band, DRAs characterized by low dielectric permittivity have been proposed in literature [129,130]. These characteristics make them suitable for high frequency applications, e.g., 30–300 GHz. Recently, some researchers utilized DRAs for biomedical applications, whose geometries and performances are discussed in the following sections.

#### 4.6.1. Techniques Employed to Enhance Radiation Characteristics of DRAs

**Figure 33:**
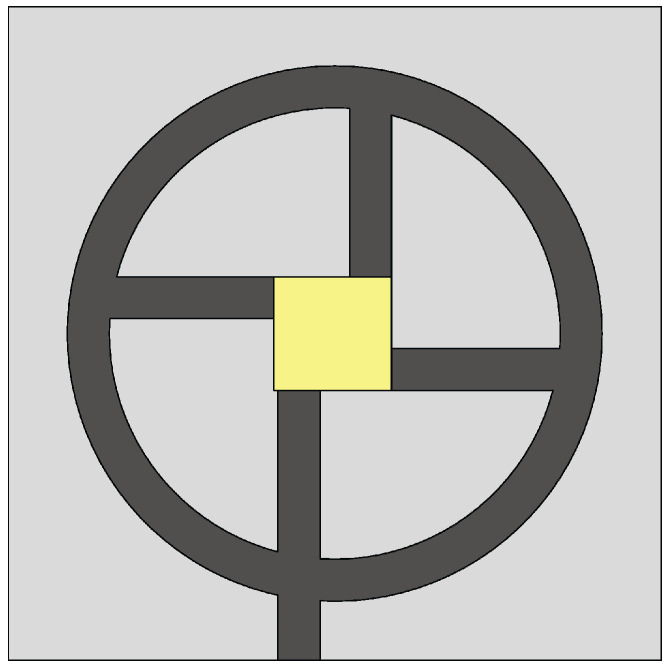
Schematic of the circular polarized DRA for biomedical applications (redrawn from [131]). Dark gray top metal face, light gray dielectric substrate, and medium yellow DRA.

An efficient way to increase the gain of DRAs is the integration with suitable structures, e.g., metal reflectors, horns, dielectric lenses, etc [128,130]. A circularly polarized DRA was designed in [131] for mm-wave microwave imaging applications. A low-cost FR-4 based DRA was utilized in this design, which can be excited using a circular loop type feed network as shown in Figure 33. This loop, with its cross-shaped arms, provides orthogonal signals in space and time to the DRA so as to excite the circular polarization. A parametric study was conducted to achieve the best possible performance. The optimized antenna has an overall size of 30 mm × 30 mm and provides an impedance bandwidth of 3 GHz from 24 GHz to 27 GHz, a constant gain of 8.6 dB in the frequency range of 25–26 GHz, and a 3 dB axial ratio (AR) bandwidth of ≈1 GHz from 25.2 GHz to 26.2 GHz.

#### 4.6.2. Techniques Used to Reduce DRAs Dimensions

**Figure 34:**
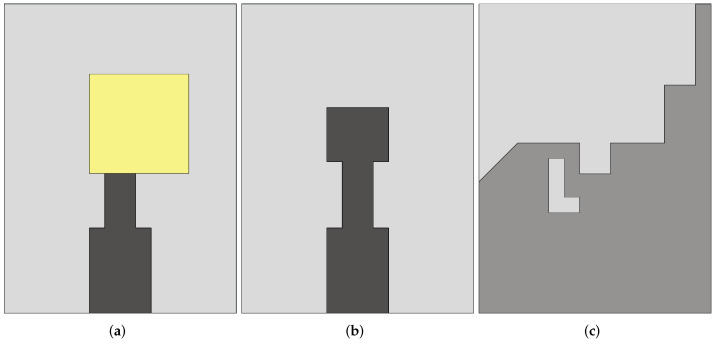
Design of a cubical shaped DRA (**a**) front-view (**b**) feed line (**c**) slotted ground plane (redrawn from [133]). Dark gray top metal face, medium gray bottom metal face, light gray dielectric substrate, and medium yellow DRA.

The size of the DRA is inversely proportional to the dielectric permittivity of the resonator. So, to decrease the size of the DRA, a material with a high dielectric permittivity can be chosen [132]. In [133], a design of a compact cubical dielectric resonator antenna (DRA), shown in Figure 34a, was presented for radar-based microwave imaging. Alumina was used to realize the DRA, which was placed on an I-shaped feed line printed on FR-4 substrate (see Figure 34b). The bottom side of the substrate is comprised of a slotted partial ground structure as shown in Figure 34c. Experimental measurements showed that the presented DRA operates in two distinct frequency ranges, namely 4.31–6.4 GHz and 9.3–13.2 GHz, where it exhibits an average simulated and measured gain of 3.88 dBi and 4.09 dBi, respectively.

A comparison among DRAs meant for biomedical applications is given in Table 7. In the table, the dimensions of the antennas are reported both as absolute values and as values normalized to the wavelength. To this end, the wavelength corresponding to the central frequency of the antenna band was considered. Although the antennas listed in the table are compact, they offer low FBW and large normalized dimensions.

## 5. Discussion

This section presents a comparison among different antenna types presented in Section 4. They are compared based on their bandwidth, radiation characteristics, design complexity, size, and cost of manufacturing. Based on these characteristics, the following remarks can be made:

- Vivaldi antennas, discussed in Section 4.1, provide wide impedance bandwidth with high gain. The designs discussed in [36,47,49,50,56,57,59] provide antennas having a wide impedance bandwidth but which are characterized by large dimensions, restricting their use in microwave imaging applications. Among the Vivaldi antennas, the designs presented in [61,62,64] are more suitable for near-field microwave imaging, as they are compact compared to other Vivaldi antennas available in the literature [58,60]. Most of these antennas operate at frequencies above 2 GHz. Designs, which operate at lower frequencies (up to 500 MHz), were presented in [37,66]. The gain of the presented Vivaldi antennas varies in the range of 8.5–11 dBi.
- Most of the planar monopole antennas presented in this paper are compact and provide wide bandwidth. Among these antennas, the designs presented in [74,77,89] show the greatest dimensions compared to other monopole designs. Most of the designs operate from 2 GHz and their gain varies in the range of 3.5–9 dBi.
- The slot antennas presented in Section 4.3 can be useful for near-field microwave imaging applications. Among these, the slot antennas presented in [100,101] offer small size, high gain, and wide impedance bandwidth.
- Most of the bowtie antennas presented in this review provide narrow bandwidth [112,117,118]. However, they are compact in size and their operating band starts at 500 MHz, thus ensuring good penetration into human tissues. The bowtie models presented in [108,113] provide UWB response and show a relatively small size compared to other bowtie antennas available in the literature [116].
- The horn antennas presented in Section 4.5 offer a large impedance bandwidth. In general, they have a high occupation volume and exhibit higher manufacturing costs than planar antennas.
- The DRA design discussed in [131] has a small size, but it operates in a frequency band that is not useful for biomedical applications. On the other hand, the DRAs show high performances that could potentially be exploited for near-field microwave imaging applications [133].

## 6. Conclusions

Microwave imaging is considered a cost-effective, highly sensitive, and safe diagnostic technique. In biomedical applications of microwave imaging, the antenna is considered the main building block, responsible for transmitting and receiving the reflected waves from the human body used for image reconstruction. Based on their application relevance, a comprehensive investigation of antennas intended for near-field microwave imaging was presented in this work. Different antenna types, such as Vivaldi antennas, planar monopole antennas, slot antennas, bowtie antennas, horn antennas, and DRAs, were discussed in detail. Among all the presented antennas, the Vivaldi antennas, planar monopole antennas, and slot antennas are considered to be good candidates for microwave imaging. Most of the presented antennas in this review generally operate in the UWB frequency range and exhibit small sizes, making them suitable to be employed in microwave imaging systems. Moreover, although they are not currently used for microwave imaging applications, wearable antennas could represent a research area on which focus the attention. In fact, if made with insulating and conductive flexible fabrics, they could facilitate the positioning of the antenna in direct contact with the body, thus favoring the transfer of electromagnetic energy inside it.

## Figures and Tables

**Table 1:** Dielectric properties of human body biological tissues at different frequencies [38].

Tissue	Frequency					
	0.5 GHz		2 GHz		5 GHz	
	$\epsilon_{r}^{\prime }$	σ	$\epsilon_{r}^{\prime }$	σ	$\epsilon_{r}^{\prime }$	σ
Skin (Dry)	44.91	0.728	38.56	1.265	35.77	3.06
Skin (Wet)	48.62	0.704	43.52	1.335	39.61	3.574
Fat	5.54	0.042	5.32	0.085	5.02	0.242
Muscle	57.32	0.843	54.16	1.508	50.13	4.24
Bone	12.94	0.10	11.65	0.31	10.04	0.962
White Matter	41	0.473	36.73	1.001	33.44	2.858
Blood	63.25	1.383	59.02	2.186	53.95	5.395

**Table 2:** Performance comparison among Vivaldi antennas designed for microwave imaging applications.

Refs.	Dimensions		Dielectric Material	Dielectric Constant	Frequency Band (GHz)	Fractional Bandwidth (%)	Peak Gain (dBi)	Operating Medium
	(mm${}^{2}$)	($\lambda^{2}$)						
[36]	44 × 74	1.05 × 1.77	Rogers 6002 & 3001	2.94 & 2.28	2.4–12	133.33	-	Air
[37]	60 × 60	0.55 × 0.55	Rogers RT6010	10.2	0.5–5	163.63	-	Coupling Medium
	60 × 60	0.55 × 0.55	T-Ceram E-20	20				
	42 × 50	0.38 × 0.45	T-Ceram E-37	37				
[45]	50 × 62	0.83 × 1.03	Taconic RF-35	3.5	2–8	120	10.4	Air
[47]	40 × 70	1.08 × 1.86	Rogers RT/Duroid 5880	2.2	4–12	100	9.8	Air
[49,50]	44 × 80	1.49 × 2.72	Rogers 6002 & 3001	2.94 & 2.28	2.4–18	153	-	Canola Oil
[56]	50.8 × 62	1.8 × 2.2	FR-4	4.4	1.3–20	175.58	10	Air
[57]	54.7 × 75	1.66 × 2.28	F4E-265	2.65	2–16.3	156.28	11	Air
[58]	150 × 150	1.25 × 1.25	FR-4	4.4	1–4	120	10.74	Air
[59]	36.3 × 59.81	0.96 × 1.58	Rogers RO3206	6.15	4.87–11	77.25	8.5	Air
[60]	75 × 88	1.06 × 1.25	Rogers RT/duroid 5870	2.33	1.54–7	127.86	9.8	Air
[61]	49 × 48.4	1.08 × 1.07	FR-4	4.3	2.9–10.4	112.78	9.58	Air
[62]	50 × 50	1.17 × 1.17	Rogers RO4003	3.38	3.1–11	112	11	Air
[63]	48 × 65.2	0.6 × 0.82	Rogers RO3003	3	2.2–5.4	84.21	-	Canola Oil
[64]	22 × 40	0.5 × 0.91	Rogers RT6010	10.2	3.1–10.6	109.48	-	Coupling Liquid
[65]	50 × 50	1.14 × 1.14	Rogers RT6010	10.2	2.75–11	120	9.4	Air
[66]	30 × 36	0.17 × 0.21	T-Ceram E-37	37	0.5–3	142.85	-	Coupling Liquid

**Table 3:** Comparative analysis among planar monopole antennas for biomedical applications.

Ref.	Dimensions		Dielectric Material	Dielectric Constant	Frequency Band (GHz)	Fractional Bandwidth (%)	Peak Gain (dBi)	Operating Medium
	(mm${}^{2}$)	($\lambda^{2}$)						
[67]	28 × 30	1.07 × 1.15	FR-4	4.4	3–20	147.82	8.81	Air
	24 × 30	0.92 × 1.15					9.08	
[68]	20 × 25	0.47 × 0.59	FR-4	4.3	3.19–11.03	110.26	6	Water
[69]	33.14 × 14.90	0.77 × 0.34	FR-4	4.4	3–11	114.28	4.74	Air
[70]	50 × 85	1.14 × 1.94	Rogers RO4003	3.38	3.1–10.6	110	9.4	Air
	-	-	Rogers RT6010	10.2				
[71]	31 × 31	0.72 × 0.72	Rogers RT/Duroid 5870	2.33	3.04–11	113.39	7.5	Air
[72]	29 × 27	0.87 × 0.81	FR-4	4.4	3–15	133.33	-	Air
[73]	22.5 × 20	0.46 × 0.41	Rogers RT6010	10.2	2.7–9.7	113	-	Air
[74]	51 × 95	0.93 × 1.74	FR-4	4.4	1–10	163.63	-	Air
[76]	23.1 × 32	0.58 × 0.8	FR-4	4.6	3.1–12	117.88	3.54	Air
[77]	50 × 50	1.08 × 1.08	FR-4	4.4	4–9	76.92	10.5	Air
[78]	10 × 10	0.21 × 0.21	Rogers RO3010	10.2	3.8–9	81.25	4	Air
[79]	12 × 18	0.35 × 0.52	FR-4	4.4	2.91–14.72	134	-	Air
[80]	12 × 18	0.35 × 0.53	FR-4	4.4	2.9–15	135	6	Air
[81]	12 × 18	0.34 × 0.51	FR-4	4.4	2.95–14.27	131.47	6	Air
[82]	12 × 18	0.31 × 0.47	FR-4	4.4	2.97–12.83	124.81	-	Air
[83]	12 × 18	0.37 × 0.56	FR-4	4.4	2.96–15.8	136.88	6.1	Air
[84]	9 × 10	0.19 × 0.21	Rogers RT6010	10.2	2.1–11	135.87	-	Air
[85]	15 × 15	0.375 × 0.375	Silicon/Benzocyclobutene	11.68/2.65	5–10	66.66	-	Oil
[86]	20 × 28	0.49 × 0.69	FR-4	4.4	2.95–12	121	4	Air
	16 × 22	0.39 × 0.54					3.6	
[87]	16 × 21	0.42 × 0.55	FR-4	4.4	3.4–12.5	114.5	5.16	Air
[88]	10.2 × 15.5	0.3 × 0.47	FR-4	4.3	4.23–14	107	5.17	Air
[89]	50 × 60	2.9 × 3.5	Denim substrate	1.4	7–28	120	10.1	Air

**Table 4:** Comparison among slot antennas meant for near-field microwave imaging.

Ref.	Dimensions		Dielectric Material	Dielectric Constant	Frequency Band (GHz)	Fractional Bandwidth (%)	Peak Gain (dBi)	Operating Medium
	(mm${}^{2}$)	($\lambda^{2}$)						
[96]	40 × 38	0.93 × 0.88	LCP	3.16	2–12	142.85	-	Air
[97]	64 × 63	1.65 × 1.62	-	2.5	2.89–12.58	125	6	Air
[98]	26 × 29	0.59 × 0.66	FR-4	4.4	3.1–10.6	109.5	-	Air
[99]	19 × 19	0.31 × 0.31	Rogers RT6010	10.2	2–8	120	-	Similar to fat
[100]	25 × 36	0.41 × 0.6	Rogers RT6010	10.2	1–9	160	12	PEG-water-solution
[101]	50 × 50	0.83 × 0.83	Rogers RT6010	10.2	1–9	160	11	PEG-water-solution
[102]	23 × 21	0.57 × 0.52	FR-4	4.4	3.1–12	117.88	6.2	Air

**Table 5:** Performance comparison among bowtie antennas designed for microwave imaging systems.

Ref.	Dimensions		Dielectric Material	Dielectric Constant	Frequency Band (GHz)	Fractional Bandwidth (%)	Peak Gain (dBi)	Operating Medium
	(mm${}^{2}$)	($\lambda^{2}$)						
[107]	18.85 × 11.62	0.77 × 0.48	-	3.2	8–16.8	70.96	8	Air
[108]	50 × 30	0.95 × 0.57	Rogers RT6010	10.2	2.5–9	113	-	$\epsilon_{r}$ = 9.5
[112]	62.5 × 62.5	0.68 × 0.68	-	9	2–4.4	75	-	Similar to fat
[113]	48 × 32	1.18 × 0.78	FR-4	4.4	3.06–11.7	117.07	8.5	Air
[116]	70.3 × 37	1.52 × 0.8	Arlon’s “Foam Clad”	1.15–1.3	3–10	107.69	-	Vegetable Oil
[117]	30 × 30	0.125 × 0.125	Rogers RT6010	10.2	0.5–2	120	7.5	PEG-water-solution
[118]	30 × 30	0.21 × 0.21	Rogers RT/Duroid 5880	2.2	0.85–3.5	121.83	-	PEG-water-solution

**Table 6:** Comparative analysis between horn antennas for microwave imaging applications.

Refs.	Length (mm)	Aperture Size		Frequency Band (GHz)	Fractional Bandwidth (%)	Peak Gain (dBi)	Operating Medium
		(mm${}^{2}$)	($\lambda^{2}$)				
[123]	70	-	-	2-12	142.85	-	Air
[124]	108	61.6 × 61.6	0.7 × 0.7	1.54-5.29	110	7.8	Canola Oil
[125,126]	75	38 × 47	0.88 × 1.09	3-11	114.28	-	Solid Dielectric
[127]	-	16 × 11	0.17 × 0.11	1.5-5	107.7	8	Air

**Table 7:** Comparative analysis among DRAs meant for near-field microwave imaging.

Ref.	Dimensions		Dielectric Material	Dielectric Constant	Frequency Band (GHz)	Fractional Bandwidth (%)	Peak Gain (dBi)	Operating Medium
	(mm${}^{2}$)	($\lambda^{2}$)						
[131]	30 × 30	2.55 × 2.55	FR-4	4.4	24–27	11.76	8.6	Air
[133]	15 × 20	0.43 × 0.58	FR-4	4.4	4.31–6.4/9.3–13.2	39.02/34.66	4.09	Air
			Alumina	9.8				

## Data Availability

Not applicable.

## References

[1] Fear E.C., Hagness S.C., Meaney P.M., Okoniewski M., Stuchly M.A. (2002). Enhancing breast tumor detection with near-field imaging. IEEE Microw. Mag..

[2] Patlak M., Nass S.J., Henderson I.C., Lashof J.C., National Research Council, Institute of Medicine, Commission on Life Sciences, National Cancer Policy Board, Committee on the Early Detection of Breast Cancer (2001). Mammography and Beyond: Developing Technologies for the Early Detection of Breast Cancer.

[3] Wells P.N. (2006). Ultrasound imaging. Phys. Med. Biol..

[4] Fear E., Stuchly M. (2000). Microwave detection of breast cancer. IEEE Trans. Microw. Theory Tech..

[5] Murphy I.G., Dillon M.F., Doherty A.O., McDermott E.W., Kelly G., O’higgins N., Hill A.D. (2007). Analysis of patients with false negative mammography and symptomatic breast carcinoma. J. Surg. Oncol..

[6] De Santis V., Sill J.M., Bourqui J., Fear E.C. (2012). Safety assessment of ultra-wideband antennas for microwave breast imaging. Bioelectromagnetics.

[7] Jofre L., Hawley M.S., Broquetas A., de Los Reyes E., Ferrando M., Elias-Fuste A.R. (1990). Medical imaging with a microwave tomographic scanner. IEEE Trans. Biomed. Eng..

[8] Bolomey J.C., Pichot C., Garboriaud G. (1991). Planar microwave imaging camera for biomedical applications: Critical and prospective analysis of reconstruction algorithms. Radio Sci..

[9] Fear E.C., Meaney P.M., Stuchly M.A. (2003). Microwaves for breast cancer detection?. IEEE Potentials.

[10] Semenov S.Y., Svenson R.H., Boulyshev A.E., Souvorov A.E., Borisov V.Y., Sizov Y., Starostin A.N., Dezern K.R., Tatsis G.P., Baranov V.Y. (1996). Microwave tomography: Two-dimensional system for biological imaging. IEEE Trans. Biomed. Eng..

[11] Meaney P.M., Fanning M.W., Li D., Poplack S.P., Paulsen K.D. (2000). A clinical prototype for active microwave imaging of the breast. IEEE Trans. Microw. Theory Tech..

[12] Catapano I., Di Donato L., Crocco L., Bucci O.M., Morabito A.F., Isernia T., Massa R. (2009). On quantitative microwave tomography of female breast. Prog. Electromagn. Res..

[13] Grzegorczyk T.M., Meaney P.M., Kaufman P.A., Paulsen K.D. (2012). Fast 3-D tomographic microwave imaging for breast cancer detection. IEEE Trans. Med. Imaging.

[14] Bond E.J., Li X., Hagness S.C., Van Veen B.D. (2003). Microwave imaging via space-time beamforming for early detection of breast cancer. IEEE Trans. Antennas Propag..

[15] Li X., Bond E.J., Van Veen B.D., Hagness S.C. (2005). An overview of ultra-wideband microwave imaging via space-time beamforming for early-stage breast-cancer detection. IEEE Antennas Propag. Mag..

[16] Fear E.C., Bourqui J., Curtis C., Mew D., Docktor B., Romano C. (2013). Microwave breast imaging with a monostatic radar-based system: A study of application to patients. IEEE Trans. Microw. Theory Tech..

[17] Hagness S.C., Taflove A., Bridges J.E. (1998). Two-dimensional FDTD analysis of a pulsed microwave confocal system for breast cancer detection: Fixed-focus and antenna-array sensors. IEEE Trans. Biomed. Eng..

[18] Klemm M., Craddock I., Leendertz J., Preece A., Benjamin R. (2008). Improved delay-and-sum beamforming algorithm for breast cancer detection. Int. J. Antennas Propag..

[19] Lim H.B., Nhung N.T.T., Li E.P., Thang N.D. (2008). Confocal microwave imaging for breast cancer detection: Delay-multiply-and-sum image reconstruction algorithm. IEEE Trans. Biomed. Eng..

[20] O’Halloran M., Glavin M., Jones E. Improved Confocal Microwave Imaging of the breast using path-dependent signal weighting. Proceedings of the 2011 XXXth URSI General Assembly and Scientific Symposium.

[21] Klemm M., Leendertz J., Gibbins D., Craddock I., Preece A., Benjamin R. (2009). Microwave radar-based breast cancer detection: Imaging in inhomogeneous breast phantoms. IEEE Antennas Wirel. Propag. Lett..

[22] Bocquet B., Van de Velde J., Mamouni A., Leroy Y., Giaux G., Delannoy J., Delvalee D. (1990). Microwave radiometric imaging at 3 GHz for the exploration of breast tumors. IEEE Trans. Microw. Theory Tech..

[23] Cheever E.A., Foster K.R. (1992). Microwave radiometry in living tissue: What does it measure?. IEEE Trans. Biomed. Eng..

[24] Kruger R.A., Kopecky K.K., Aisen A.M., Reinecke D.R., Kruger G.A., Kiser W.L. (1999). Thermoacoustic CT with radio waves: A medical imaging paradigm. Radiology.

[25] He X., Geyi W., Wang S. (2015). A hexagonal focused array for microwave hyperthermia: Optimal design and experiment. IEEE Antennas Wirel. Propag. Lett..

[26] Chen Z., Sun H., Geyi W. (2017). Maximum wireless power transfer to the implantable device in the radiative near field. IEEE Antennas Wirel. Propag. Lett..

[27] Cicchetti R., Faraone A., Testa O. (2018). Energy-based representation of multiport circuits and antennas suitable for near-and far-field syntheses. IEEE Trans. Antennas Propag..

[28] Cicchetti R., Faraone A., Testa O. (2019). Near field synthesis based on multi-port antenna radiation matrix eigenfields. IEEE Access.

[29] Rumsey V. Frequency independent antennas. Proceedings of the 1958 IRE International Convention Record.

[30] Collin R.E. (2007). Foundations for Microwave Engineering.

[31] Shlivinski A., Heyman E., Kastner R. (1997). Antenna characterization in the time domain. IEEE Trans. Antennas Propag..

[32] Sörgel W., Wiesbeck W. (2005). Influence of the antennas on the ultra-wideband transmission. EURASIP J. Adv. Signal Process..

[33] Sörgel W., Pivit F., Wiesbeck W. Comparison of frequency domain and time domain measurement procedures for ultra wideband antennas. Proceedings of the 25th Annual Meeting and Symposium of the Antenna and Measurement Techniques Association (AMTA’03).

[34] Baum C.E., Farr E.G. (1993). Impulse radiating antennas. Ultra-Wideband, Short-Pulse Electromagnetics.

[35] Quintero G., Zurcher J.F., Skrivervik A.K. (2011). System fidelity factor: A new method for comparing UWB antennas. IEEE Trans. Antennas Propag..

[36] Bourqui J., Okoniewski M., Fear E.C. Balanced antipodal Vivaldi antenna for breast cancer detection. Proceedings of the Second European Conference on Antennas and Propagation.

[37] Wang M., Crocco L., Cavagnaro M. (2021). On the Design of a Microwave Imaging System to Monitor Thermal Ablation of Liver Tumors. IEEE J. Electromagn. RF Microwaves Med. Biol..

[38] https://www.fcc.gov/general/body-tissue-dielectric-parameters.

[39] Gibson P. The Vivaldi aerial. Proceedings of the 1979 9th European Microwave Conference.

[40] Gross F.B. (2011). Frontiers in Antennas: Next Generation Design & Engineering.

[41] Perdana M., Hariyadi T., Wahyu Y. (2017). Design of vivaldi microstrip antenna for ultra-wideband radar applications. IOP Conf. Ser. Mater. Sci. Eng..

[42] Cicchetti R., Cicchetti V., Faraone A., Foged L., Testa O. (2021). A compact high-gain wideband lens vivaldi antenna for wireless communications and through-the-wall imaging. IEEE Trans. Antennas Propag..

[43] Janaswamy R., Schaubert D. (1987). Analysis of the tapered slot antenna. IEEE Trans. Antennas Propag..

[44] Sugawara S., Maita Y., Adachi K., Mori K., Mizuno K. Characteristics of a mm-wave tapered slot antenna with corrugated edges. Proceedings of the 1998 IEEE MTT-S International Microwave Symposium Digest (Cat. No. 98CH36192).

[45] Abbak M., Akıncı M., Çayören M., Akduman İ. (2017). Experimental microwave imaging with a novel corrugated Vivaldi antenna. IEEE Trans. Antennas Propag..

[46] Gazit E. (1988). Improved design of the Vivaldi antenna. IEE Proc. H (Microwaves, Antennas Propag.).

[47] Kanjaa M., El Mrabet O., Khalladi M., Essaaidi M. Exponentially tapered antipodal Vivaldi antenna for breast cancer detection. Proceedings of the 2015 IEEE 15th Mediterranean Microwave Symposium (MMS).

[48] Langley J., Hall P., Newham P. (1993). Novel ultrawide-bandwidth Vivaldi antenna with low cross polarisation. Electron. Lett..

[49] Bourqui J., Campbell M.A., Williams T., Fear E.C. (2010). Antenna evaluation for ultra-wideband microwave imaging. Int. J. Antennas Propag..

[50] Bourqui J., Okoniewski M., Fear E.C. (2010). Balanced antipodal Vivaldi antenna with dielectric director for near-field microwave imaging. IEEE Trans. Antennas Propag..

[51] Sharma N., Bhatia S.S. (2020). Performance enhancement of nested hexagonal ring-shaped compact multiband integrated wideband fractal antennas for wireless applications. Int. J. RF Microw. Comput.-Aided Eng..

[52] Ataeiseresht R., Ghobadi C., Nourinia J. (2006). A novel analysis of Minkowski fractal microstrip patch antenna. J. Electromagn. Waves Appl..

[53] Sundaram A., Maddela M., Ramadoss R. (2007). Koch-fractal folded-slot antenna characteristics. IEEE Antennas Wirel. Propag. Lett..

[54] Vinoy K., Jose K., Varadan V., Varadan V. Resonant frequency of Hilbert curve fractal antennas. Proceedings of the IEEE Antennas and Propagation Society International Symposium. 2001 Digest. Held in conjunction with: USNC/URSI National Radio Science Meeting (Cat. No. 01CH37229).

[55] Romeu J., Soler J. (2001). Generalized Sierpinski fractal multiband antenna. IEEE Trans. Antennas Propag..

[56] Biswas B., Ghatak R., Poddar D. (2017). A fern fractal leaf inspired wideband antipodal Vivaldi antenna for microwave imaging system. IEEE Trans. Antennas Propag..

[57] Zhao C., Li X., Yang M., Sun C. (2021). Resistance-loaded miniaturized dual-layer Vivaldi antenna for plasma reflection diagnosis. Microw. Opt. Technol. Lett..

[58] Nurhayati N., De Oliveira A.M., Justo J.F., Setijadi E., Sukoco B.E., Endryansyah E. (2020). Palm tree coplanar Vivaldi antenna for near field radar application. Microw. Opt. Technol. Lett..

[59] de Oliveira A.M., Justo J.F., Perotoni M.B., Kofuji S.T., Neto A.G., Bueno R.C., Baudrand H. (2017). A high directive Koch fractal Vivaldi antenna design for medical near-field microwave imaging applications. Microw. Opt. Technol. Lett..

[60] Islam M.T., Mahmud M.Z., Misran N., Takada J.I., Cho M. (2017). Microwave breast phantom measurement system with compact side slotted directional antenna. IEEE Access.

[61] Guruswamy S., Chinniah R., Thangavelu K. (2019). A printed compact UWB Vivaldi antenna with hemi cylindrical slots and directors for microwave imaging applications. AEU—Int. J. Electron. Commun..

[62] Abbosh A.M. (2008). Directive antenna for ultrawideband medical imaging systems. Int. J. Antennas Propag..

[63] Zhang J., Fear E.C., Johnston R.H. (2009). Cross-Vivaldi antenna for breast tumor detection. Microw. Opt. Technol. Lett..

[64] Mohammed B.J., Abbosh A.M., Bialkowski M.E. Design of tapered slot antenna operating in coupling liquid for ultrawideband microwave imaging systems. Proceedings of the 2011 IEEE International Symposium on Antennas and Propagation (APSURSI).

[65] Abbosh A., Kan H., Bialkowski M. (2006). Compact ultra-wideband planar tapered slot antenna for use in a microwave imaging system. Microw. Opt. Technol. Lett..

[66] Molaei A., Dagheyan A.G., Juesas J.H., Martinez-Lorenzo J. Miniaturized UWB Antipodal Vivaldi Antenna for a mechatronic breast cancer imaging system. Proceedings of the 2015 IEEE International Symposium on Antennas and Propagation & USNC/URSI National Radio Science Meeting.

[67] Eesuola A., Chen Y., Tian G. Novel ultra-wideband directional antennas for microwave breast cancer detection. Proceedings of the 2011 IEEE International Symposium on Antennas and Propagation (APSURSI).

[68] Ojaroudi M., Civi Ö.A. High efficiency loop sleeve monopole antenna for array based UWB microwave imaging systems. Proceedings of the 2016 IEEE International Symposium on Antennas and Propagation (APSURSI).

[69] Danjuma I.M., Akinsolu M.O., See C.H., Abd-Alhameed R.A., Liu B. (2020). Design and optimization of a slotted monopole antenna for ultra-wide band body centric imaging applications. IEEE J. Electromagn. RF Microwaves Med. Biol..

[70] Pittella E., Bernardi P., Cavagnaro M., Pisa S., Piuzzi E. (2011). Design of UWB antennas to monitor cardiac activity. ACES J.—Appl. Comput. Electromagn. Soc..

[71] Mahmud M., Islam M.T., Samsuzzaman M. (2016). A high performance UWB antenna design for microwave imaging system. Microw. Opt. Technol. Lett..

[72] Subramanian S., Sundarambal B., Nirmal D. (2018). Investigation on simulation-based specific absorption rate in ultra-wideband antenna for breast cancer detection. IEEE Sens. J..

[73] Kanj H., Popovic M. (2005). Miniaturized microstrip-fed “Dark Eyes" antenna for near-field microwave sensing. IEEE Antennas Wirel. Propag. Lett..

[74] Lee D., Nowinski D., Augustine R. (2018). A UWB sensor based on resistively-loaded dipole antenna for skull healing on cranial surgery phantom models. Microw. Opt. Technol. Lett..

[75] Wu T., King R. (1965). The cylindrical antenna with nonreflecting resistive loading. IEEE Trans. Antennas Propag..

[76] Islam M., Islam M., Samsuzzaman M., Faruque M., Misran N. (2015). Microstrip line-fed fractal antenna with a high fidelity factor for UWB imaging applications. Microw. Opt. Technol. Lett..

[77] Golezani J.J., Abbak M., Akduman I. (2012). Modified directional wide band printed monopole antenna for use in radar and microwave imaging applications. Prog. Electromagn. Res..

[78] Ahadi M., Isa M.B.M., Saripan M.I.B., Hasan W.Z.W. (2014). Square monopole antenna for microwave imaging, design and characterisation. IET Microwaves Antennas Propag..

[79] Halili K., Ojaroudi M., Ojaroudi N. (2012). Ultrawideband monopole antenna for use in a circular cylindrical microwave imaging system. Microw. Opt. Technol. Lett..

[80] Ojaroudi N., Ghadimi N. (2015). Omnidirectional microstrip monopole antenna design for use in microwave imaging systems. Microw. Opt. Technol. Lett..

[81] Ojaroudi N., Ojaroudi M., Ebazadeh Y. (2014). UWB/omni-directional microstrip monopole antenna for microwave imaging applications. Prog. Electromagn. Res..

[82] Ojaroudi N., Ojaroudi M., Ghadimi N. (2012). UWB omnidirectional square monopole antenna for use in circular cylindrical microwave imaging systems. IEEE Antennas Wirel. Propag. Lett..

[83] Abdollahvand A., Pirhadi A., Ebrahimian H., Abdollahvand M. (2014). A compact UWB printed antenna with bandwidth enhancement for in-body microwave imaging applications. Prog. Electromagn. Res..

[84] Abbosh A., Bialkowski M. (2009). Compact directional antenna for ultra wideband microwave imaging system. Microw. Opt. Technol. Lett..

[85] Woten D.A., El-Shenawee M. (2008). Broadband dual linear polarized antenna for statistical detection of breast cancer. IEEE Trans. Antennas Propag..

[86] Lasemi Z., Atlasbaf Z. (2020). Impact of Fidelity Factor on Breast Cancer Detection. IEEE Antennas Wirel. Propag. Lett..

[87] Islam M., Islam M., Samsuzzaman M., Faruque M. (2015). A negative index metamaterial antenna for UWB microwave imaging applications. Microw. Opt. Technol. Lett..

[88] Afifi A., Abdel-Rahman A.B., Allam A., Abd El-Hameed A.S. A compact ultra-wideband monopole antenna for breast cancer detection. Proceedings of the 2016 IEEE 59th International Midwest Symposium on Circuits and Systems (MWSCAS).

[89] Mahmood S.N., Ishak A.J., Saeidi T., Soh A.C., Jalal A., Imran M.A., Abbasi Q.H. (2021). Full Ground Ultra-Wideband Wearable Textile Antenna for Breast Cancer and Wireless Body Area Network Applications. Micromachines.

[90] Yoshimura Y. (1972). A microstripline slot antenna (short papers). IEEE Trans. Microw. Theory Tech..

[91] Kahrizi M., Sarkar T.K., Maricevic Z.A. (1993). Analysis of a wide radiating slot in the ground plane of a microstrip line. IEEE Trans. Microw. Theory Tech..

[92] Das B., Prasad K. (1984). Impedance of a transverse slot in the ground plane of an offset stripline. IEEE Trans. Antennas Propag..

[93] Jang Y.W. (2003). A wideband and high gain microstrip four-slot antenna array. Microw. J..

[94] Chen X., Huang K., Xu X.B. (2011). A novel planar slot array antenna with omnidirectional pattern. IEEE Trans. Antennas Propag..

[95] Chen C., Li C., Zhu Z., Wu W. (2017). Wideband and low-cross-polarization planar annual ring slot antenna. IEEE Antennas Wirel. Propag. Lett..

[96] Tavassolian N., Nikolaou S., Tentzeris M.M. A flexible UWB elliptical slot antenna with a tuning uneven U-shape stub on LCP for microwave tumor detection. Proceedings of the 2007 Asia-Pacific Microwave Conference.

[97] Hossain I., Noghanian S., Shafai L., Pistorius S. (2009). Coplanar waveguide fed taper arc slot antenna for microwave imaging and ultra wide band applications. Microw. Opt. Technol. Lett..

[98] See T.S., Chen Z., Qing X. Proximity effect of UWB antenna on human body. Proceedings of the 2009 Asia Pacific Microwave Conference.

[99] Wang Y., Fathy A.E., Mahfouz M.R. Novel compact tapered microstrip slot antenna for microwave breast imaging. Proceedings of the 2011 IEEE International Symposium on Antennas and Propagation (APSURSI).

[100] Li X., Yan J., Jalilvand M., Zwick T. A compact double-elliptical slot-antenna for medical applications. Proceedings of the 2012 6th European Conference on Antennas and Propagation (EUCAP).

[101] Li X., Sit Y.L., Zwirello L., Zwick T. (2013). A miniaturized UWB stepped-slot antenna for medical diagnostic imaging. Microw. Opt. Technol. Lett..

[102] Islam M.T., Samsuzzaman M., Rahman M., Islam M. (2018). A compact slotted patch antenna for breast tumor detection. Microw. Opt. Technol. Lett..

[103] Compton R., McPhedran R., Popovic Z., Rebeiz G., Tong P., Rutledge D. (1987). Bow-tie antennas on a dielectric half-space: Theory and experiment. IEEE Trans. Antennas Propag..

[104] Stutzman W.L., Thiele G.A. (2012). Antenna Theory and Design.

[105] Balanis C.A. (2011). Modern Antenna Handbook.

[106] Bailey M. (1984). Broad-band half-wave dipole. IEEE Trans. Antennas Propag..

[107] Eldek A.A., Elsherbeni A.Z., Smith C.E. Wideband bow-tie slot antenna with tuning stubs. Proceedings of the 2004 IEEE Radar Conference (IEEE Cat. No. 04CH37509).

[108] Shannon C.J., Fear E., Okoniewski M. (2005). Dielectric-filled slotline bowtie antenna for breast cancer detection. Electron. Lett..

[109] Kumar A., Hristov H.D. (1989). Microwave Cavity Antennas.

[110] Li R., Thompson D., Tentzeris M.M., Laskar J., Papapolymerou J. (2005). Development of a wide-band short backfire antenna excited by an unbalance-fed H-shaped slot. IEEE Trans. Antennas Propag..

[111] Qu S.W., Li J.L., Xue Q., Chan C.H. (2008). Wideband cavity-backed bowtie antenna with pattern improvement. IEEE Trans. Antennas Propag..

[112] Yun X., Fear E.C., Johnston R.H. (2005). Compact antenna for radar-based breast cancer detection. IEEE Trans. Antennas Propag..

[113] Kwag Y.K., Hassanein A.D., Edwards D.J. (2009). A high-directive bowtie radar antenna with a pyramidal reflector for ultra wideband radar imaging applications. Microw. Opt. Technol. Lett..

[114] Lestari A.A., Yarovoy A.G., Ligthart L.P. (2004). RC-loaded bow-tie antenna for improved pulse radiation. IEEE Trans. Antennas Propag..

[115] Lestari A.A., Bharata E., Suksmono A.B., Kurniawan A., Yarovoy A.G., Ligthart L.P. (2010). A modified bow-tie antenna for improved pulse radiation. IEEE Trans. Antennas Propag..

[116] See C.H., Abd-Alhameed R.A., Chung S.W.J., Zhou D., Al-Ahmad H., Excell P.S. (2012). The design of a resistively loaded bowtie antenna for applications in breast cancer detection systems. IEEE Trans. Antennas Propag..

[117] Li X., Jalilvand M., Sit Y.L., Zwick T. (2014). A compact double-layer on-body matched bowtie antenna for medical diagnosis. IEEE Trans. Antennas Propag..

[118] Jalilvand M., Vasanelli C., Wu C., Kowalewski J., Zwick T. On the evaluation of a proposed bowtie antenna for microwave tomography. Proceedings of the 8th European Conference on Antennas and Propagation (EuCAP 2014).

[119] Love A.W. (1976). Electromagnetic Horn Antennas.

[120] Russo P., Rudduck R., Peters L. (1965). A method for computing E-plane patterns of horn antennas. IEEE Trans. Antennas Propag..

[121] Yu J., Rudduck R., Peters L. (1966). Comprehensive analysis for E-plane of horn antennas by edge diffraction theory. IEEE Trans. Antennas Propag..

[122] Hamid M. (1968). Diffraction by a conical horn. IEEE Trans. Antennas Propag..

[123] Campbell M.A., Okoniewski M., Fear E.C. (2010). TEM horn antenna for near-field microwave imaging. Microw. Opt. Technol. Lett..

[124] Latif S.I., Flores Tapia D., Rodriguez Herrera D., Solis Nepote M., Pistorius S., Shafai L. (2015). A directional antenna in a matching liquid for microwave radar imaging. Int. J. Antennas Propag..

[125] K Amineh R., Trehan A., Nikolova N.K. (2009). TEM horn antenna for ultra-wide band microwave breast imaging. Prog. Electromagn. Res..

[126] Amineh R.K., Ravan M., Trehan A., Nikolova N.K. (2010). Near-field microwave imaging based on aperture raster scanning with TEM horn antennas. IEEE Trans. Antennas Propag..

[127] di Clemente F.S., Stephan R., Hein M. Ultra-wideband miniaturised high permittivity-matched antennas for biomedical diagnostic. Proceedings of the 2013 7th European Conference on Antennas and Propagation (EuCAP).

[128] Baldazzi E., Al-Rawi A., Cicchetti R., Smolders A.B., Testa O., van Coevorden Moreno C.d.J., Caratelli D. (2020). A high-gain dielectric resonator antenna with plastic-based conical horn for millimeter-wave applications. IEEE Antennas Wirel. Propag. Lett..

[129] Simeoni M., Cicchetti R., Yarovoy A., Caratelli D. (2011). Plastic-based supershaped dielectric resonator antennas for wide-band applications. IEEE Trans. Antennas Propag..

[130] Cicchetti R., Faraone A., Miozzi E., Ravanelli R., Testa O. (2016). A high-gain mushroom-shaped dielectric resonator antenna for wideband wireless applications. IEEE Trans. Antennas Propag..

[131] Singhwal S.S., Kanaujia B.K., Singh A., Kishor J. (2019). Novel circularly polarized dielectric resonator antenna for microwave image sensing application. Microw. Opt. Technol. Lett..

[132] Keyrouz S., Caratelli D. (2016). Dielectric resonator antennas: Basic concepts, design guidelines, and recent developments at millimeter-wave frequencies. Int. J. Antennas Propag..

[133] Kaur G., Kaur A. (2021). Monostatic radar-based microwave imaging of breast tumor detection using a compact cubical dielectric resonator antenna. Microw. Opt. Technol. Lett..

